# Spatial and single-cell transcriptome analysis reveals changes in gene expression in response to drug perturbation in rat kidney

**DOI:** 10.1093/dnares/dsac007

**Published:** 2022-03-23

**Authors:** Naoki Onoda, Ayako Kawabata, Kumi Hasegawa, Megumi Sakakura, Itaru Urakawa, Masahide Seki, Junko Zenkoh, Ayako Suzuki, Yutaka Suzuki

**Affiliations:** 1 Department of Computational Biology and Medical Sciences, Graduate School of Frontier Sciences, The University of Tokyo, Kashiwa-shi, Chiba 277-0882, Japan; 2 Research Core Function Laboratories, Research Unit, R&D Division, Kyowa Kirin Co., Ltd., Machida-shi, Tokyo 194-8533, Japan; 3 Biomedical Science Research Laboratories 1, Research Unit, R&D Division, Kyowa Kirin Co., Ltd., Machida-shi, Tokyo 194-8533, Japan

**Keywords:** spatial transcriptome, single-cell transcriptome, kidney, rat, losartan

## Abstract

The kidney is a complex organ that consists of various types of cells. It is occasionally difficult to resolve molecular alterations and possible perturbations that the kidney experiences due to drug-induced damage. In this study, we performed spatial and single-cell transcriptome analysis of rat kidneys and constructed a precise rat renal cell atlas with spatial information. Using the constructed catalogue, we were able to characterize cells of several minor populations, such as macula densa or juxtaglomerular cells. Further inspection of the spatial gene expression data allowed us to identify the upregulation of genes involved in the renin regulating pathway in losartan-treated populations. Losartan is an angiotensin II receptor antagonist drug, and the observed upregulation of the renin pathway-related genes could be due to feedback from the hypotensive action of the drug. Furthermore, we found spatial heterogeneity in the response to losartan among the glomeruli. These results collectively indicate that integrated single-cell and spatial gene expression analysis is a powerful approach to reveal the detailed associations between the different cell types spanning the complicated renal compartments.

## 1. Introduction

The kidney is a highly complex organ that exhibits various functions, including the excretion of wastes, maintenance of water, mineral, or metabolite balance, and regulation of blood pressure.^1^^,^^2^ To accomplish such diverse functions, various types of cells are present in different compartments of the kidney, performing a coordinated function in each. For example, tubule-glomerular feedback occurs as a result of the fabricated coordination among multiple cell types through a mechanism where the specialized cells of the thick ascending limb (TAL), macula densa, sense the luminal concentration of electrolytes and alter the vascular pressure to adapt glomerular filtration rate (GFR) via the juxtaglomerular (JG) cells.^3^ The tubule-glomerular feedback system is utilized to maintain the GFR, thus playing a pivotal role in the response to alterations of blood pressure caused by both endogenous and exogenous factors. Hypertension is associated with decreased GFR and it is a common diagnostic index for chronic kidney disease (CKD), which impairs the quality of life, increases the risk of other diseases such as cardiovascular diseases, and ultimately requires treatment with a high burden, such as kidney transplantation or dialysis.^4^^,^^5^ Angiotensin II type 1 receptor (AT1) blocker (ARB) has been widely used to treat CKD with hypertension, as it exhibits hypotensive action and inhibits the renin–angiotensin system.^6^ Losartan is the first clinically used ARB and still plays an important role in basic and clinical research as a prototypic molecule to elucidate the potential effects of other ARBs.^7^ However, even for such an established drug, the mechanism by which it achieves kidney protection is not thoroughly understood, and consequently the processes involving the complicated cellular system of the kidney.^8^ In fact, despite substantial potential clinical relevance, the molecular mechanisms of kidney failure have not been clearly elucidated.^9^ Our current knowledge still mainly arises from the rodent models, namely, rats or mice.^10^ However, even in model animals, little is known about the detailed molecular mechanisms that occur in the kidney at spatial and single-cell resolutions.

Single-cell RNA sequencing (scRNA-seq) has been rapidly developing and is utilized to uncover cellular heterogeneity in several research fields.^11^ Although there are various platforms developed for scRNA-seq, these platforms generally require the prior isolation of single cells. The cell dissociation procedures such as homogenization, enzymatic treatment, or flow cytometry, often cause biases in the profiles of the collected cells. In the case of the kidney, even differences in temperature during enzymatic dissociation affect the final proportion of the cell types and the gene expression profile.^12^ Most importantly, it results in the loss of spatial information of the cells, as several cells have a specific role characterized by their spatial positioning, such as the macula densa. As obtaining spatial information is difficult, spatial transcriptome sequencing (ST-seq) is a newly developed approach for quantitating spatial gene expression from the pathological sections.^13^ Unlike scRNA-seq, ST-seq does not require single-cell isolation; thus, it is possible to quantify gene expression without introducing a bias during cell isolation. ST-seq has made a substantial impact on several research fields, such as cancer or neurology.^14^^,^^15^ In particular, since its initial launch, the Visium system of 10× Genomics has been widely used as an easy and commercially available ST-seq platform. Despite the general success of ST-seq, the resolution of the present Visium system has not yet reached the single-cell level. This drawback imposes a substantial barrier to the application of ST-seq to inherently complex organs, such as kidneys, other than analyses of brains or cancers, where a larger-scale spatial expression pattern beyond the single cell has been the main research target. The integration of both scRNA-seq and ST-seq data is essential for complementing the insufficient spatial resolution by computational approaches.

A limited number of studies have used these techniques to construct a renal cell atlas in mice.^16–19^ However, there have been fewer studies,^20^^,^^21^ particularly pharmacological studies, in the rat rodent model. Moreover, in these studies, the primary data came from scRNA-seq, resulting in the loss of spatial information. Therefore, limited information is presented on the location of individual cells in the context of their tissue compartment, including histopathological features of each cell and possible cell–cell interactions with adjacent cells, although only two studies using the ST-seq dataset were recently reported in mice for the characterization of some murine disease models.^22^^,^^23^ Spatial information on each cell is particularly important when the response of the whole kidney to a particular drug must be understood, where the cellular function may be different depending on the spatial context. In this study, we simultaneously conducted ST-seq and scRNA-seq analyses of rat kidneys to comprehensively characterize the transcriptome features of all renal cells including their spatial coordination to create a spatial renal cell atlas. We further described the local transcriptional responses against losartan and the heterogeneous response to the drug among the glomeruli in the complicated tissue structures of the kidneys. Our study proposed a conceptual flow to evaluate the detailed drug response at the single-cell and spatial resolutions.

## 2. Materials and methods

### 2.1. Animals and losartan treatment

Six-week-old male Sprague Dawley rats from Charles River were orally administered 20 mg/kg losartan potassium (Tokyo Chemical Industry; L0232) once daily for 2 days. Twenty-four hours after the final administration, the rats were euthanized, and their kidneys were collected for ST-seq or scRNA-seq analysis. All animal studies were performed in accordance with the Standards for Proper Conduct of Animal Experiments at Kyowa Kirin Co., Ltd., under the approval of the company’s Institutional Animal Care and Use Committee. Tokyo Research Park of Kyowa Kirin Co., Ltd. is fully accredited by AAALAC International.

### 2.2. Library preparation for ST-seq

The kidneys from the rats were horizontally divided into three pieces, and each central part was further divided into two pieces. Each piece of the kidney was embedded in Optimal Cutting Temperature Compound (Sakura Finetek Japan; 4583) and immediately fresh frozen on dry ice. Frozen tissue sections of 10 µm thickness were sliced (Leica CM1950) and affixed to the slides for Visium spatial sequencing. The sequencing libraries were prepared using the Visium Spatial Gene Expression Slide & Reagents Kit (10× Genomics; 1000187), following the manufacturer’s instructions (10× Genomics; CG000239_Rev_A). The histological images of each sample were acquired using an inverted microscope (Nikon Eclipse Ti). In this study, the tissue sections were permeabilized for 18 min, defined by the tissue optimization flow performed in advance, using the Visium Spatial Tissue Optimization Slide & Reagents Kit (10× Genomics; 1000193). The libraries were sequenced using Illumina NovaSeq.

### 2.3. Tissue dissociation for scRNA-seq

Rat kidneys were dissociated mechanically and enzymatically for scRNA-seq. The central part of each kidney was vertically sectioned, and the slices were subsequently dissociated by shaking for 60 min at 37°C in Dulbecco’s Modified Eagle Medium (Nacalai; 08456-36), containing 1.6 mg/ml collagenase (Sigma; C2674) and 5 U DNaseI (Takara; 2270A). The resultant was then filtered using a 40-µm cell strainer (Falcon; 352340). The cell suspension was washed by density gradient centrifugation with Optiprep (AXS, 1114524) and 1% (w/v) Bovine Serum Albumin containing Phosphate-Buffered Saline (Nacalai; 09968-35), followed by filtering using a 35-µm cell strainer (Falcon; 352235).

### 2.4. Library preparation for scRNA-seq

For each sample, the quantity and viability of the cells were confirmed, and a lack of aggregated cells or debris was microscopically observed. The scRNA-seq libraries were prepared using the Chromium Single Cell 3’ Library Kit v2 (10× Genomics; 120237) and i7 Multiplex Kit (10× Genomics; 120262), following the manufacturer’s instructions (CG000075_Rev_C). The libraries were sequenced using Illumina NovaSeq.

### 2.5. Data processing for ST-seq datasets

Sequencing results (BCL files) were converted into FASTQ files and mapped to the rat reference genome (Rat.Rnor6.ensembl; http://igenomes.illumina.com.s3-website-us-east-1.amazonaws.com/Rattus_norvegicus/Ensembl/Rnor_6.0/Rattus_norvegicus_Ensembl_Rnor_6.0.tar.gz, 28 March 2022, date last accessed) using Space Ranger (v.1.1.0; 10× Genomics; https://support.10xgenomics.com/spatial-gene-expression/software/downloads/1.1/, 28 March 2022, date last accessed). The downstream analysis was performed using the R package, Seurat (v. 3.2.2; https://satijalab.org/seurat/articles/install.html, 28 March 2022, date last accessed). The outputs from Space Ranger were converted into a Seurat object and pre-processed using the SCTransform function.^24^ The objects from each sample were integrated using the Seurat anchor-based integration workflow,^25^ followed by principal component analysis, dimensionality reduction, and unsupervised clustering.

### 2.6. Data processing for scRNA-seq datasets

Sequencing results (BCL files) were converted into FASTQ files and mapped to the rat reference genome (Rat.Rnor6.ensembl) using Cell Ranger (v.4.0.0; 10× Genomics; https://support.10xgenomics.com/single-cell-gene-expression/software/downloads/4.0/, 28 March 2022, date last accessed). The downstream analysis was performed using the R package, Seurat (v. 3.2.2). The output matrices from Cell Ranger were converted into Seurat objects and quality control was performed to retain the cells with 200–5,000 genes, under 30,000 total Unique Molecular Identifier, and fewer than 40% of the mitochondrial reads. The objects from each sample were preprocessed using the Seurat standard workflow with the functions of NormalizeData and FindVariableFeatures, and subsequently integrated by the Seurat anchor-based integration workflow^25^ followed by principal component analysis, dimensionality reduction, and unsupervised clustering. The three major subsets, epithelial, immune, and stromal, were identified based on unsupervised clustering and known marker gene expression. Each subset was separately subjected to principal component analysis, dimensionality reduction, and unsupervised clustering.

### 2.7. Integration of ST-seq and scRNA-seq datasets

The integration of ST-seq and scRNA-seq datasets was performed following the Seurat anchor-based integration workflow.^25^ For the integration, every major subset identified during scRNA-seq dataset processing was separately supplied as a reference. Consequently, the prediction score of each cell type was calculated for every single spot in the ST-seq datasets.

### 2.8. Cross-species comparison of gene expression

For comparative analyses, the gene names in the rat ST-seq or scRNA-seq datasets were converted to the homologous genes in humans or mice by referring to the corresponding tables acquired from Ensembl biomart (https://www.ensembl.org/biomart/martview/0a21ad93aa1acd892a848e74de5d0d24, 28 March 2022, date last accessed). We used the ST-seq (GSE171406) and scRNA-seq (GSE107585; mouse, GSE151302) dataset which is available from the NCBI Gene Expression Omnibus with the corresponding accession number. The histological regions of murine ST-seq were determined in a similar way as the original paper,^23^ referring to the histological image and the result of a Graph-based cluster calculated using Spaceranger. The cell types in both human and murine scRNA-seq datasets are publicly available.

### 2.9. Gene ontology enrichment analysis

Gene ontology (GO) enrichment analysis was performed using Metascape^26^ (https://metascape.org/gp/index.html#/main/step1, 28 March 2022, date last accessed) with default parameters. The top 20 highly enriched terms are shown as bar graphs.

### 2.10. Deconvolution analysis of spatial datasets

The deconvolution of each spot in the spatial datasets was performed using CIBBERSORTx^27^ (https://cibersortx.stanford.edu/, 28 March 2022, date last accessed) Cell Fraction module with default parameters. The expression matrix with annotated cell type labels of each major population from a scRNA-seq dataset was separately used as a reference sample and that of the entire ST-seq dataset was used as the input mixture sample. Consequently, the cell fraction of each spot was imputed.

### 2.11. Normalization of expression levels with imputed fraction

The normalization of gene expression patterns with a specific cell type was calculated using the following equations:

$${\mathrm{NC}}_{ij}=\frac{C_{ij}}{F_{j}},$$

where NC is the normalized count matrix of *n* genes and *m* spots, *C* is the corresponding count matrix after the correction by SCTransform,^24^ and *F* is the vector of the fraction for the cell type in interest, imputed by CIBERSORTx.^27^ For every *i* gene and *j* spot, NC was calculated, followed by further normalization with total count using the NormalizeData function in Seurat.

### 2.12. Statistical analysis and data availability

Statistical analyses were performed using R version 3.6.1. Fisher’s exact tests were used to compare the spatial distribution of TALs and the response to losartan of the glomeruli. Wilcoxon’s rank sum test was used to compare gene expression between groups. Pearson’s correlation analysis was used to compare the gene expression between rats and humans or mice. The Kolmogorov–Smirnov test was used to determine whether the gene expression levels followed a Gaussian distribution. The degree of skew for each gene expression distribution was calculated to observe the heterogeneity of their expression patterns. All sequencing data that support the findings of this study have been deposited in the DNA Data Bank of Japan with the accession number DRA012650. The histological images for ST-seq data have also been deposited in Genomic Expression Archive with the accession number E-GEAD-450. The processed data of scRNA-seq and ST-seq are also provided in the database DBKERO (https://kero.hgc.jp/, 28 March 2022, date last accessed).

## 3. Results

### 3.1. Spatial transcriptomics reveals complicated renal structures in rat

**Figure 1:**
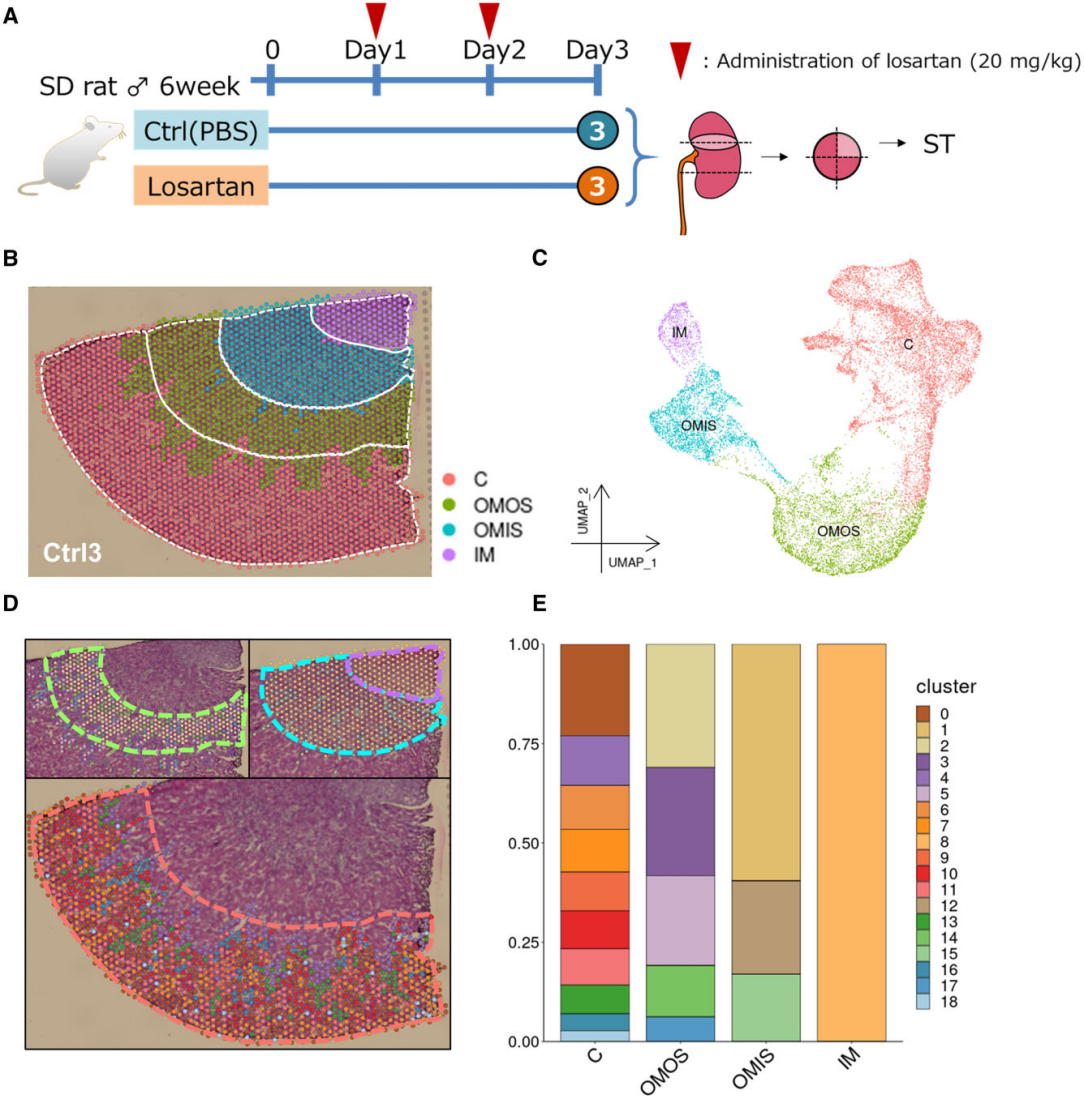
ST analysis of rat kidney. (A) A schematic image of the experimental design used in this study. (B, C) The spots detected in a control rat on its histological images (B) and a representation of the UMAP for dimension reduction (C) with the whole samples integrated, coloured by the histological annotation. C, cortex; OMOS, outer medulla outer stripe; OMIS, outer medulla inner stripe; IM, inner medulla, made by assigning each unsupervised cluster into four histological annotations based on their position. (D) The spots coloured by unsupervised clustering on histological images. Each histological annotation, Cortex (bottom), OMOS (left top), or OMIS and IM (right top), is separately shown via a broken line in each panel. (E) The proportion of the total 20 unsupervised clusters in each histological annotation.

We performed ST-seq analysis of the kidneys, which were collected from rats with or without administration of 20 mg/kg/day losartan (Fig. 1A). At this dose, relevant increases in blood renin and angiotensin levels have previously been observed in rats.^28^ For ST-seq analysis, tissue sections including both cortical and medullary regions of the kidney were prepared. In each section, four histological regions of the kidney structure were identified and annotated as the cortex, outer medulla outer stripe (OMOS), outer medulla inner stripe (OMIS), and inner medulla (IM), by a specialized histologist (Fig. 1B and Supplementary Fig. S1A). A total of 16,428 spots were obtained from three control and three losartan-treated rats, and a median of 4,950 genes was detected from each spot (Supplementary Table S1). Together, 19 clusters were obtained as a result of unsupervised clustering (Supplementary Fig. S1B). Each cluster was assigned to one of four histological annotation labels based on their histological positioning (Fig. 1B and C). Even though the clustering analysis was performed solely based on their transcriptome information, the obtained clusters were consistent with the histologically defined positions (Fig. 1B) and well separated in the Uniform Manifold Approximation and Projection (UMAP) plot (Fig. 1C).

To validate the reproducibility of the obtained datasets, we compared the results between triplicated ST-seq datasets obtained from losartan-treated rats and control rats. The results were generally highly reproducible (Supplementary Fig. S1A–C). When we further inspected the possible cause of the lack of reproducibility, we found that reproducibility was particularly lower for the IM in the Ctrl rat 1. We also found that the outer region contained a large number of clusters with a higher clustering resolution (Fig. 1D and E and Supplementary Fig. S1B). In fact, out of the total 19 identified clusters, 15 clusters were located in the outer regions, of which 10 clusters were located in the cortex and 5 clusters were located in the OMOS. In contrast, the OMIS region contained three clusters and the IM contained only one cluster. These observations may be derived from the fact that cellular density is generally higher in the medullar region, and each spot tends to be homogeneous as a mixture of many types of cells. The spatial resolution of OMIS or IM region may be lower than that of the cortical or OMOS region; thus, it may have caused the lower reproducibility, depending on the stochastic overlapping pattern of the ST-seq spot over the complex cellular components, as well as manipulation problems during sectioning. Otherwise, aside from the exceptional cases which we excluded from our following analyses, we did not observe any obvious differences in the histopathological and transcriptomic features between losartan-treated and control rats in our analysis (Supplementary Fig. S1A–D; see also below).

In addition, we compared the rat gene expression with that of human and mouse, using the publicly available ST-seq datasets.^23^ The human ST-seq dataset only included the cortical region, thus we compared the gene expression of the spots from the corresponding region in the normal rat datasets. Consequently, the expression profile of the present rat dataset had a high correlation with the human dataset (Supplementary Fig. S1E). Regarding the mouse dataset, we compared the spots from every region; the cortex (C), OMOS, OMIS, and IM, with the sham mouse and in the normal rat datasets. Unlike the result from human, the mouse cortex region showed a relatively low correlation with that of the rat but OMOS or OMIS and IM showed a high correlation (Supplementary Fig. S1F).

### 3.2. Single-cell transcriptomics reveals various types of cells in the rat kidney

**Figure 2:**
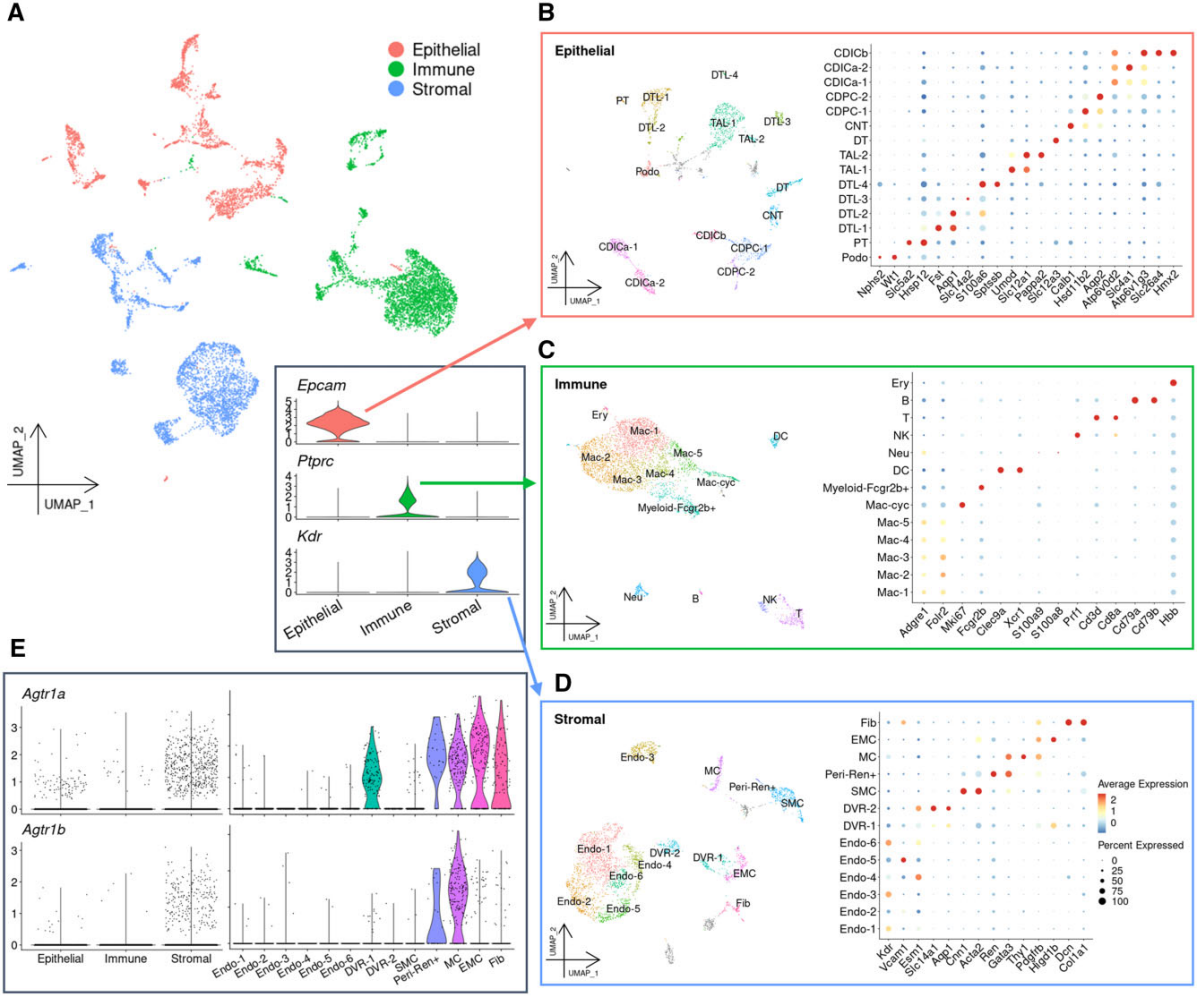
High-resolution characterization of rat kidney cells using single-cell RNA-seq. (A) Projection of UMAP dimensionality reduction coloured by the three main populations: epithelial, immune, and stromal, as identified by unsupervised clustering and annotation with marker genes. Expression of the marker genes for each population is shown in the panel at the bottom right. (B–D) Projection of UMAP dimensionality reduction (left) and the results of annotation with the marker genes (right) of each population. The dots coloured grey in UMAP projections show contamination with cells from other populations. In the dot plots for the annotation results, the colour of each dot shows the scaled expression levels of the marker genes for each cell type, and the size of each dot shows the percentage of the expressing cells in each cell type. In the Epithelial population (B), the following cell types were identified: Podo, podocyte; PT, proximal tubule; DTL, descending thin limb; TAL, thick ascending limb; DT, distal tubule; CNT, connecting tubule; CDPC, collecting duct principal cell; CDICa, collecting duct intercalated cell type A; CDICb, collecting duct intercalated cell type B. In the Immune population (C), the following cell types were identified: Mac, macrophage; Mac-cyc, proliferating macrophage; Myeloid-Fcgr2b+, *Fcgr2b*-expressing myeloid; DC, dendritic cell; Neu, neutrophil; NK, natural killer cell; T, T cell; B, B cell; Ery, erythrocyte. In the Stromal population (D), the following cell types were identified: Endo, endothelial cell; DVR, descending vasa recta; SMC, smooth muscle cell; Peri-Ren+, *Ren*-expressing pericyte; MC, mesangial cell; EMC, extraglomerular mesangial cell; Fib, fibroblast. (E) Expression of the genes encoding angiotensin II receptor type 1 for each main population (left) and for each cell type of the Stromal population.

To complement the lack of resolution of the ST-seq data, we generated scRNA-seq data from kidney cells of rats treated using the same conditions as those used for the ST-seq analysis. As a result, 14,830 cells were collected from control and losartan-treated rats (Supplementary Table S2). A median of 1,140 genes was detected in each cell. There are three major known populations, representing the nephric cellular groups ‘Epithelial’, ‘Immune’, and other ‘Stromal’ cells (Fig. 2A). We inspected the sub-populations of these three populations depending on the expression patterns of cell type markers^1^^,^^29–33^ (Fig. 2B–D).

In the ‘Epithelial’ cell population, all cell types constituting the nephron segment were represented (Fig. 2B). Cells of the nephron segments, such as the TAL or descending thin limb (DTL) in the loop of Henle (LOH) were further divided into multiple minor sub-populations. Notably, DTL-1 also expressed a marker gene for proximal tubule (PT), thus DTL-1 could also include a medullary part of PT which localizes in OMOS.^33^ Furthermore, the expression profiles of DTL-2 and DLT-3 were consistent with the previously characterized expression profiles of juxtamedullary and cortical nephron,^16^ respectively. Similarly, from the collecting duct, principal cell (CDPC) and intercalated cell type A (CDICa) were also divided into two sub-populations. In the ‘Immune’ cell population, macrophages and other myeloid cells composed a major cluster (Fig. 2C). Six sub-populations of macrophages were characterized based on the expression patterns of the common macrophage markers *Adgre1*, *Folr2*, and *C1qc* (Fig. 2C and Supplementary Fig. S2A). Myeloid cells were recognized by the gene expression of an Fc receptor, *Fcgr2b*. Cell clusters of dendritic cells, neutrophils, natural killer cells, T cells, and B cells were identified, and erythrocytes are represented as a minor population. In the third ‘Stromal’ cell population, we found a major population of endothelial cells which included six sub-populations defined by endothelial markers *Kdr* or *Vcam1* (Fig. 2D). Two clusters represented the descending vasa recta (DVR), mainly defined by the urea transporter *Slc14a1*. Furthermore, other stromal cells, such as smooth muscle cells, renin-expressing pericytes (Peri-Ren+), mesangial cells (MC), extraglomerular mesangial cells (EMC) and fibroblasts were also represented (Fig. 2D). Additionally, the specifically expressed genes for each cell type annotated above were calculated (Supplementary Table S3). These cell populations were consistent with previous estimations. The obtained fine gene expression patterns provided an essential basis for analysing the spatial gene expression patterns represented by the ST-seq data. Of note, *Agtr1a* and *Agtr1b*, which are genes encoding the main targets of losartan, AT1, were expressed in several stromal cell sub-populations (Fig. 2E). Consistent with previous reports, Peri-Ren+ and MC were found to express both *Agtr1a* and *Agtr1b*. However, a sub-population of DVR, EMC, and fibroblasts expressed only *Agtr1a*^34^ (Fig. 2E, right). In the following analysis, we focussed on cells that play major roles in losartan response.

Furthermore, we also performed a cross-species comparison using the murine^1^ (Supplementary Fig. S3A) and human^32^ scRNA-seq (Supplementary Fig. S3B) dataset. We compared the expression profiles from mouse nephric epithelial cells to those from the corresponding cells of the present rat dataset. The information for the previous annotation has been publicly available, but the detailed definitions of cell types were slightly different from this study, e.g. the LOH in mouse includes both DTL and TAL in rat, CD-IC in mouse includes both CDICa and CDICb in rat, and the murine DCT is defined as DT in this study. Subsequently, although we observed high correlations between mice and rats across most of the cell types, only podocytes showed a relatively low correlation.

### 3.3. Integration of ST-seq with scRNA-seq for spatial characterization of kidney cells

**Figure 3:**
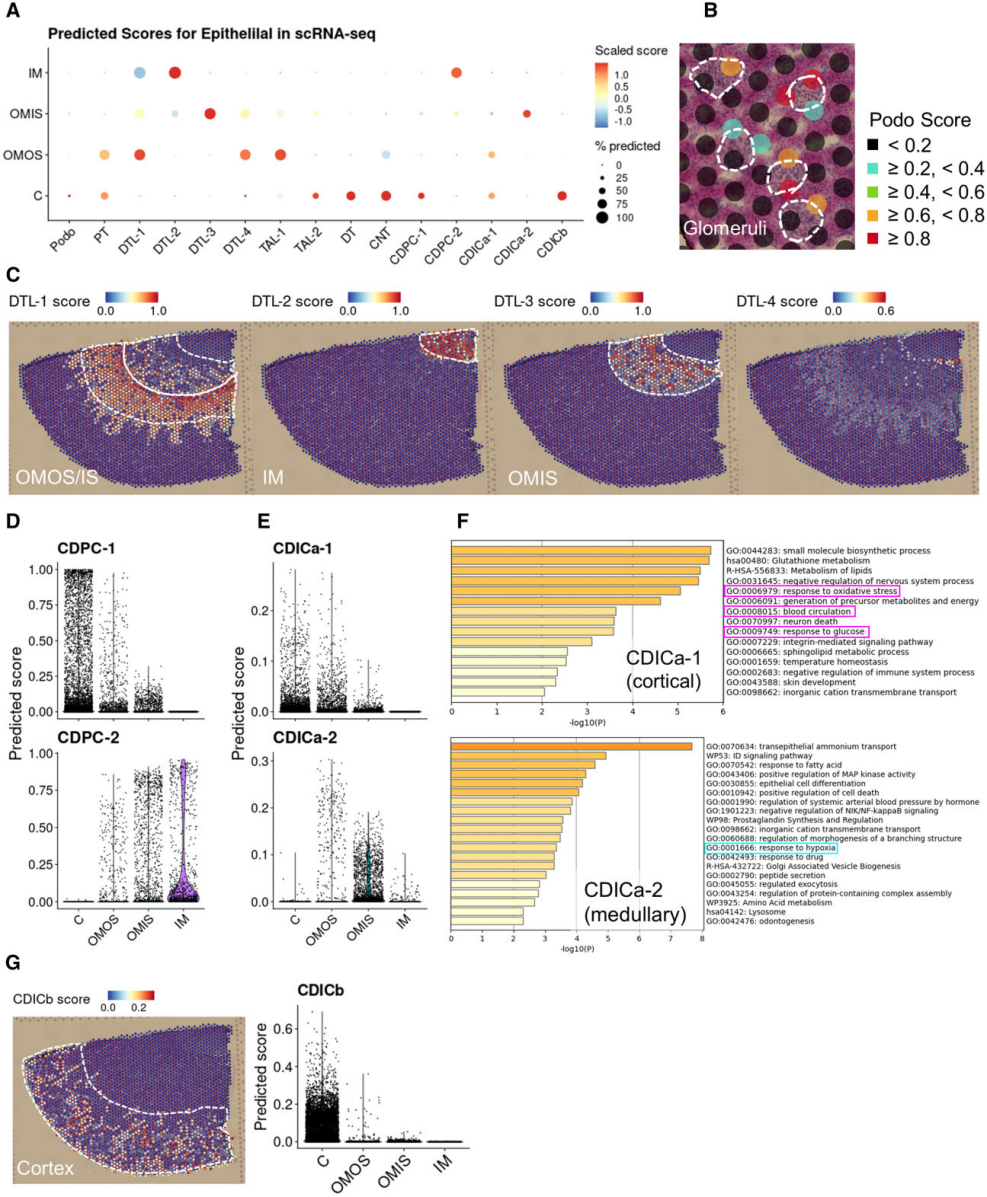
Integration of spatial and single-cell transcriptome information of rat kidneys. (A) Predicted scores of each cell type in the Epithelial population from the scRNA-seq data (*x*-axis) for each histological annotation in the ST-seq data (*y*-axis). The colour of the dots represents the scaled predicted scores, and the size of the dots represents the percentage of the spots with a predicted score larger than 0 in each histological annotation. (B) Predicted scores for podocytes of each spot on a section from a control rat. Dashed white circles show the regions histologically annotated as glomeruli. (C) The distribution of the predicted scores for the four sub-populations of the descending thin limb on a histological image of a control rat. Dashed white lines show the corresponding histological annotations. (D, E) The distribution of the predicted scores for the sub-populations of CDPC (D) and intercalated cells type A (E) in each histological annotation. (F) The results of GO enrichment analysis using the differentially expressed genes between the sub-populations in CDICa. The *x*-axis of each bar graph shows the *P*-value with negative log10 conversion. (G) The distribution of the predicted scores for the sub-populations of collecting duct intercalated cells type B on a histological image of a control rat, with the dashed white line indicating the Cortex annotated area (left), and in each histological annotation (right).

To integrate the collected scRNA-seq data with ST-seq data, we conducted an anchor-based integration analysis.^25^ Using this approach, the probable existence of each cell type was imputed in each spot as predicted scores. For reference expression information, the three main populations in the scRNA-seq data (Fig. 2) were utilized independently. As expected, each spot represents transcriptome patterns from a mixture of several cell types considering the fact that multiple cell types were assigned to each cluster (Supplementary Fig. S4A). In the ‘Epithelial’ cell population, we found that podocyte, PT, distal tubule (DT), and connecting tubule populations were highly enriched in the cortical area of ST-seq data, which is consistent with previous knowledge on their spatial localization (Fig. 3A). In fact, the spots that histologically overlapped with glomeruli exhibited a high podocyte score (Fig. 3B). Notably, podocytes score was specifically enriched in ST-seq cluster 10 (ST-seq data; Fig. 1) in the cortical region (Supplementary Fig. S4A), indicating that this cluster of ST-seq data represents glomeruli. Out of the four DTL populations, the three larger populations (DTL-1; PT and DTL in juxtamedullary nephron, DTL-2; DTL in juxtamedullary nephron and DTL-3; DTL in cortical nephron) were spatially restricted in the OMOS-OMIS, IM, and OMIS regions, respectively (Fig. 3A and C and Supplementary Fig. S4B). In addition, cell type-specific genes for each sub-population (calculated by scRNA-seq; Supplementary Table S4) were expressed in regions with high prediction scores of the corresponding cell type (Supplementary Fig. S4C).

We identified distinct spatial distributions of the CDPC (Fig. 3D) and CDICa (Fig. 3E) sub-populations. These cells are known to be arranged in a mosaic pattern at a collecting duct,^35^^,^^36^ and the differences in their spatial positioning have not been well characterized. As a result of the integration analysis, although CDPC-1 and CDICa-1 were located in the cortical region, CDPC-2 and CDICa-2 were located in the inner regions. Similar to the DTL sub-populations, a series of differentially expressed genes were found between the two sub-populations (calculated based on the scRNA-seq: Supplementary Table S5; the regions giving their imputation scores were identified by the ST-seq data: Supplementary Fig. S5A and B). We subjected these differentially expressed genes between CDICa sub-populations to GO enrichment analysis.^26^ We found that GO terms including ‘response to oxidative stress’, ‘blood circulation’, and ‘response to glucose’ were associated with the cortical sub-population (CDICa-1, upper in Fig. 3F). Conversely, the GO term ‘response to hypoxia’ was associated with the medullary sub-population (CDICa-2, lower in Fig. 3F). These results indicate the possibility that the medullary region resided in a more ischaemic condition, which has been considered based on the renal anatomy.^37^ In addition, the GO term ‘transepithelial ammonium transport’ was listed with the highest *P*-value for the medullary sub-population. CDICa has a role to reabsorb ammonium excreted from TAL mainly through Na+-K+-ATPase whose efficiency is known to be affected by the ambient ammonium concentration. As the intestinal ammonium concentration is higher in the medullar region than the cortical region, the ammonium uptake by Na+-K+-ATPase is critical for IMCD ammonium secretion.^38^ Furthermore, we compared the expression profiles of the present CDPC sub-populations to the spatially distinct expression patterns of CDPC previously characterized by single nucleus RNA-seq (snRNA-seq) from humans with experimental zonation.^39^ As a result, we observed that the spatially distinct expression patterns in humans were mostly in agreement with the present datasets from rat, particularly in the medullar sub-population (Supplementary Fig. S5C). Of note, the predicted scores for CDICb were enriched at the cortical region only (Fig. 3A and G and Supplementary Fig. S5D).

Integration analysis was also performed for the ‘Immune’ cell populations (Supplementary Fig. S4D). We again found that the spatial distribution of macrophage sub-populations was highly heterogeneous. Three of the clusters (Mac-3, 4, and 5) were enriched in the OMIS region, while Mac-1 was enriched in the OMOS and cortex. However, another cluster, Mac-2, was specifically found in the cortex region. Regarding the ‘Stromal’ cell populations, four endothelial clusters (Endo-1, 2, 3, and 6) were found in both cortex and OMOS regions. On the other hand, Endo-4 and Endo-5 were biased to the OMOS and OMIS regions respectively (Supplementary Fig. S4E). In addition, two DVR sub-populations were separated into the OMOS/OMIS and OMIS/IM regions.

### 3.4. Characterization of the local transcriptomic alterations along with the renin–angiotensin axis in response to losartan

**Figure 4:**
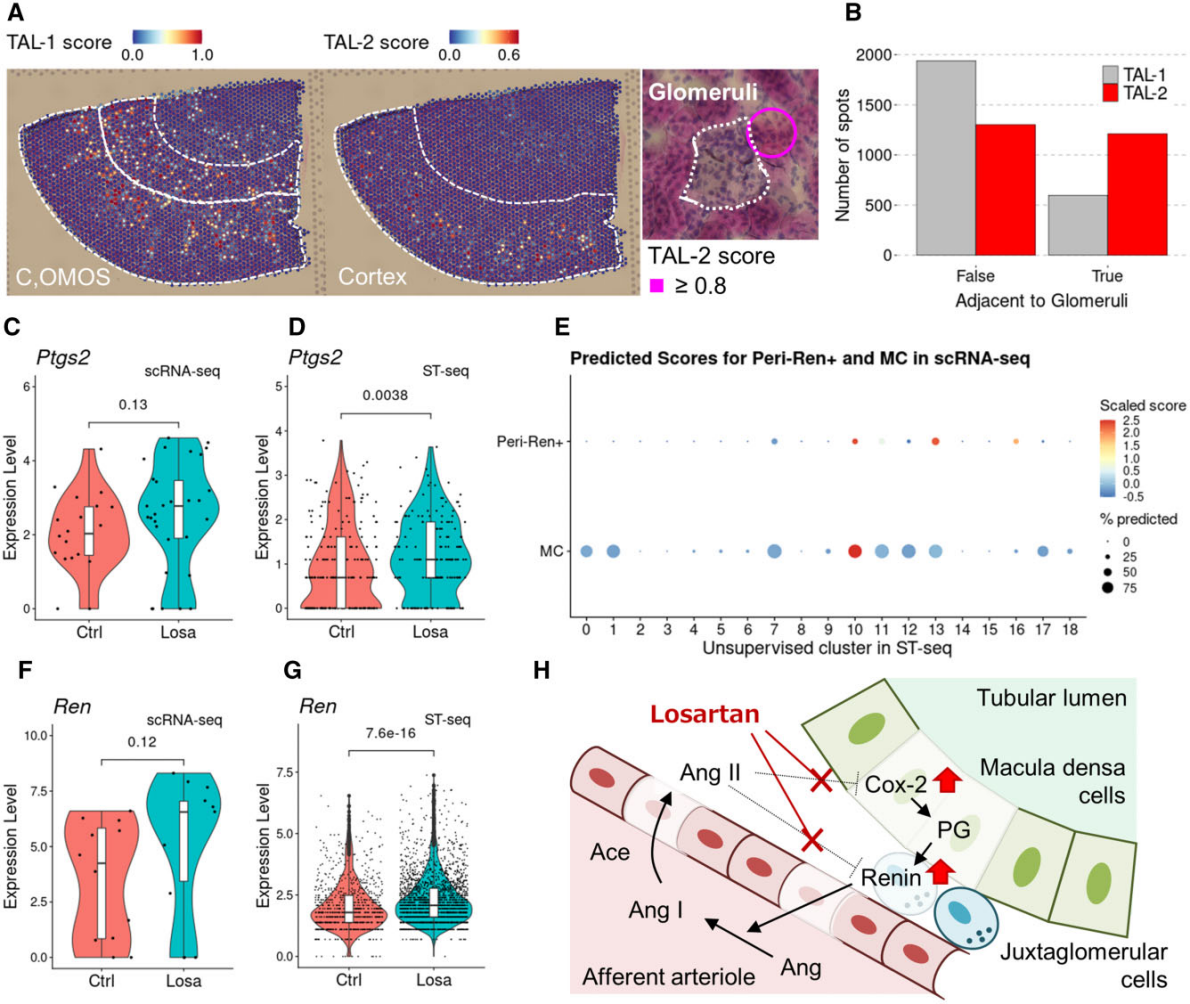
Expression patterns of macula densa and Ren-positive JG cells. (A) The distribution of the predicted scores for the two sub-populations of TAL (left), and the spot around a glomerulus with a high TAL-2 score (circle; right) on a histological image of a control rat. Dashed lines show the corresponding histological annotations or glomerulus, respectively. (B) The cortical spots in which predicted scores for TAL-1 or TAL-2 were larger than 0 were classified by the TAL-1 and TAL-2 scores. In cases where the TAL-1 score was higher than the other, the spot was classified into the TAL-1 group. The number of spots adjacent to the spots annotated as glomeruli was shown for each classified group, TAL-1 or TAL-2. (C, D) Expression of *Ptgs2* in the TAL-2 sub-population in scRNA-seq data (C), and the spots selected based on the imputed TAL-2 fraction in ST-seq data (D) for a rat treated with losartan (Losa) or not (Ctrl). (E) Predicted scores of Peri-Ren+ and MC sub-populations in the Stromal population from the scRNA-seq data (*y*-axis) for each unsupervised cluster in the ST-seq data (*x*-axis). The colour of the dots represents the scaled predicted scores, and the size of the dots represents the percentage of the spots with a predicted score larger than 0 in each histological annotation. (F, G) Expression of the *Ren* gene in the Peri-Ren+ sub-population in scRNA-seq data (F), and the cortical spots selected based on the imputed Peri-Ren+ fraction in ST-seq data (G) for the Losa or Ctrl groups. Statistical analysis was performed by Wilcoxon rank sum test, and calculated *P*-values are shown. (H) A schematic illustration of the mode of action for losartan observed in this study. Losartan is a competitive antagonist for the receptor of angiotensin II (Ang II) and exhibits hypotensive action via the inhibition of the renin–angiotensin system in the circulation. Ang II normally represses the expression of Cox-2, encoded by the *Ptgs2* gene, in macula densa cells. Cox-2 is an essential enzyme for the synthesis of Prostaglandins (PG). PG produced in macula desnsa cells induce the expression of Renin, encoded by the *Ren* gene, in JG cells and which is normally repressed by Ang II in a negative feedback system. Renin plays an important role in the renin–angiotensin system to exhibit hypertensive action. In this study, the increased expression of the *Ptgs2* gene in macula densa cells and the *Ren* gene in JG cells after the administration of losartan were observed, as shown by  upward block arrows in the figure.

We first attempted to characterize hitherto insufficiently annotated cells using the constructed transcriptome catalogue of the rat kidney. In particular, we attempted to resolve the potentially heterogeneous effects of losartan treatment within the complicated renal structure. First, we characterized the TAL sub-populations. Although high TAL-1 scores were observed over a wide region of the cortex and OMOS, those for TAL-2 were specifically observed in a restricted part of the cortex region, particularly adjacent to a glomerulus (Fig. 4A and Supplementary Fig. S6A and B). We quantified spatial heterogeneity in TAL sub-populations and found that spots with higher TAL-2 scores were predominantly located in a region adjacent to the glomeruli (Supplementary Table S6 and Fig. 4B). These results collectively indicate that this TAL-2 sub-population corresponds to the macula densa cells. These cells are unique among TAL, are localized adjacent to a glomerulus, and are known to play an important role in the cellular feedback interactions between the tubule-glomerular regions.^3^ Consistently, a gene reported as macula densa cell markers, Pappa2,^40^ was observed among genes specifically expressed in the TAL-2 cells (Supplementary Fig. S6C and Table S7). Macula densa cells play a particularly important role in the response to losartan. In macula densa cells, ARB, such as losartan, is designed to block the binding of angiotensin II to its cognitive receptor and thereby regulate expression of cyclooxygenase-2 (Cox-2), encoded by *Ptgs2*.^41^ When we examined the gene expression of the presumed macula densa population cell in the scRNA-seq dataset, we found that the expression level of *Ptgs2* appeared to be higher in the losartan-treated rats than in the control rats, although its difference was not statistically relevant (Fig. 4C). In fact, *Ptgs2* itself was identified as a differentially expressed gene with the sixth largest fold change in expression levels (Supplementary Fig. S6D).

Despite several circumstantial indications, there was still a concern that the observed changes truly represented the gene expression changes in macula densa cells. Generally, since each spot from the ST-seq data collectively represents the expressions of a mixture of several cell types, it is not always easy to directly capture the expression change in a small population of the cell within a given spot. To address this concern in a more quantitative manner with the help of bioinformatics, we conducted deconvolution analysis for each spot using CIBERSORTx.^27^ For this purpose, we utilized scRNA-seq data as the reference vectors. We particularly focussed on the 374 macula densa spots, which showed a relevant level of the calculated TAL-2 fraction (Supplementary Fig. S6E and F). Using this analysis, we confirmed a significantly higher expression of *Ptgs2* in the presumed macula densa spots in the rats treated with losartan than in those from the control rats (Fig. 4D). In addition, we further calculated the differentially expressed genes in the macula densa spots using the expression data normalized to the estimated fraction for macula densa cells of each spot. We found that several genes that are reported to play important roles in macula densa cells, including *Slc12a1* encoding NKCC2, were upregulated (Supplementary Fig. S6G). Notably, the upregulation of this gene may further indicate that the level of tubular electrolytes changes.^42^ Based on this evidence, we concluded that the TAL-2 cells were mainly composed of macula densa cells, and the gene expression changes in these cells were correctly represented by the combination of scRNA-seq and ST-seq data.

We next focussed on Peri-Ren+ cells, which are pericytes that express renin itself. The prediction score of this cell sub-population was higher in the ST-seq spots included in cluster 10, which were mostly localized at the glomeruli, similar to the score of MC-like cells, which are well known to localize to the glomeruli (Fig. 4E). Considering its spatial localization in glomeruli and renin expression, thereby identifying and imputing the cellular sub-population, Peri-Ren+, should be JG cells, which are the centre of the losartan analysis, since these cells are the major sources of renin. Figure 4H illustrates the known cascade for the mode of action of ARB, including losartan. The inhibition of angiotensin II in macula densa cells promotes the synthesis of prostaglandins via Cox-2. Subsequently, prostaglandins promote the expression of renin, which is encoded by the *Ren* gene, in JG cells. Consistent with this scheme, we found that the expression level of renin appeared to be higher in losartan-treated rats than in controls in the scRNA-seq data (Fig. 4F and Supplementary Fig. S7A). Similar to the case of the macula densa cells, we identified a subset of the JG cells/spots that showed a relevant level of the Peri-Ren+ fraction from the cortical region (Supplementary Fig. S7B and C). Consequently, we found that *Ren* expression in the JG cells/spots was substantially higher in rats treated with losartan than in the control rats (Fig. 4G). Of note, even in the presumed glomeruli spots that are included in the ST-seq cluster 10, a significantly higher expression of the *Ren* gene was observed in response to losartan (Supplementary Fig. S7D).

### 3.5. Heterogeneity in the glomerular responses to losartan treatment

**Figure 5:**
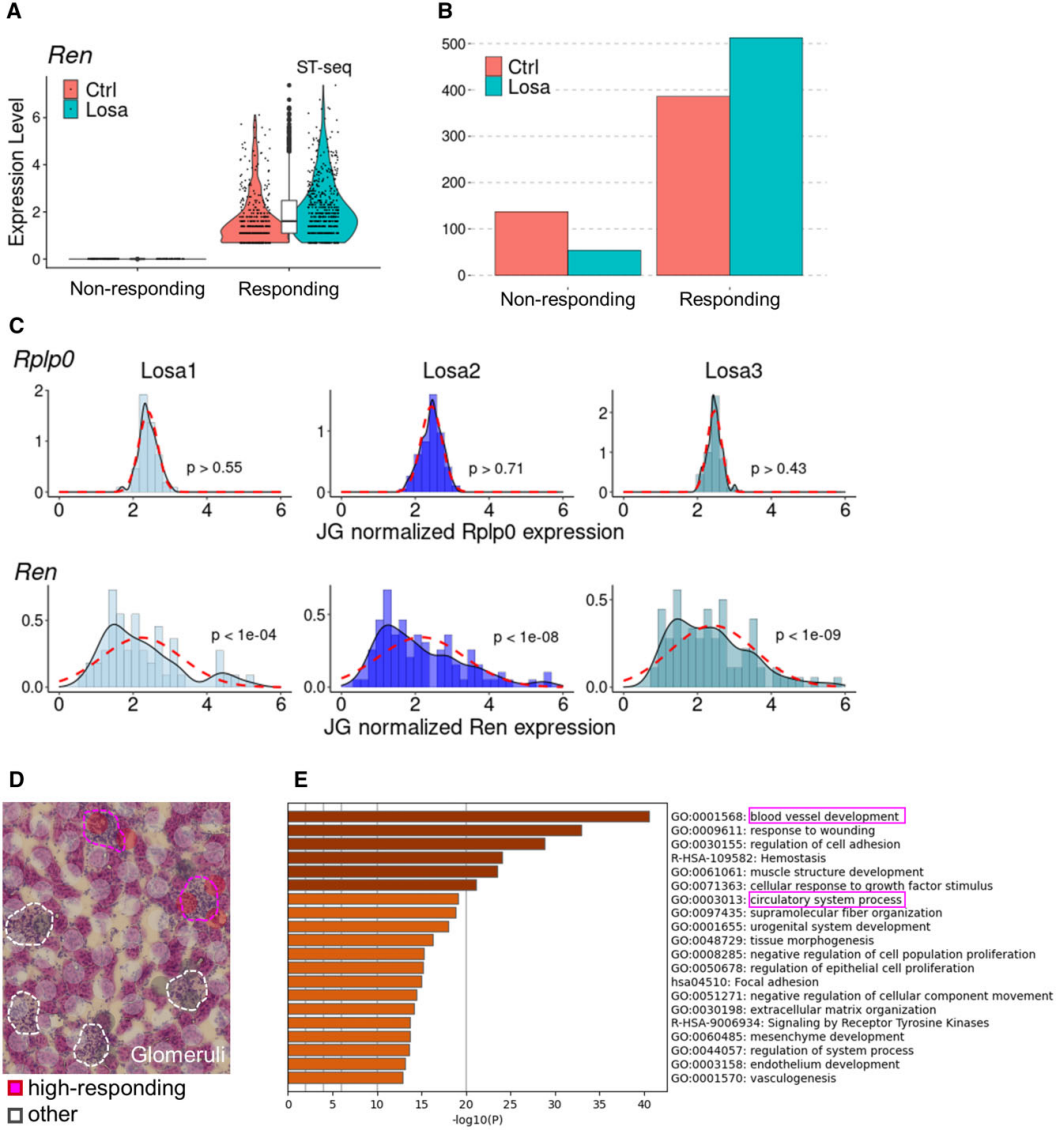
Heterogeneity in the response to losartan among the glomeruli. (A) Expression of the *Ren* gene in selected spots overlying the glomeruli is shown, separated by the experimental groups losartan treated (Losa) or not (Ctrl). The selected spots were classified into two groups: Responding or Non-responding to losartan, based on the expression of the *Ren* gene. (B) The number of the spots in the Responding and Non-responding groups is shown, separated by Losa or Ctrl. (C) The distribution of a housekeeping gene, *Rplp0*, (upper) and the *Ren* gene (bottom) with expression levels normalized using the imputed fraction of JG cells from each sample, followed by further normalization with the total count of each cell and log1p conversion. Dashed lines indicate the Gaussian distribution calculated with the average and standard deviation of each sample. The *P*-values from Kolmogorov–Smirnov test for each sample to the corresponding Gaussian distribution are shown. (D) The ‘high-responding’ or other spots on a section from a rat treated with losartan. Histologically defined glomeruli with high-responding or other spots are shown by dashed circles. (E) The results of GO enrichment analysis using the differentially expressed genes between the high-responding glomeruli spots and the others. The *x*-axis of each bar graph shows the *P*-value with negative log10 conversion.

During the course of the above analyses, we noticed that the expression levels exhibited some spatial diversity, even among representative spots of the same cellular or structural category. We wondered if this observation may represent the fact that there should be a particular heterogeneity, even among the glomeruli. In particular, there is a certain heterogeneity in the renin–angiotensin axis, as shown in Fig. 4H, depending on individual glomeruli; thus, the induction level of the gene expression changes in response to losartan may differ. To further analyse this possibility, we generated a subset of the spots that histologically overlapped with the glomeruli (Supplementary Fig. S8A). Essentially, most of the selected spots were confirmed to belong to the presumed glomeruli cluster, cluster 10 (Supplementary Fig. S8B). We classified the spots according to the induction rate of *Ren* gene expression (Fig. 5A). As expected, a significantly higher number of responding spots were found in the losartan-treated rats than in the control rats (Fig. 5B and Supplementary Table S8).

To more precisely compare the levels of the response among these glomeruli, we further selected a group of spots that contained a certain level of JG cell fraction. The degree of the fraction was calculated by deconvolution analysis using CIBERSORTx^27^ (Supplementary Fig. S8C). To further remove the potential noise derived from the inherently different number of cells included in each spot, we normalized the count expression of the *Ren* gene, dividing by the calculated JG cell fraction of each spot. Even after careful data processing, we found that there was a group of JG spots showing more intensive responses to the losartan treatment. More precisely, the distribution of *Ren* gene expression did not significantly follow the Gaussian distribution as a random response model even after normalization, whereas housekeeping genes, such as the 60S acidic ribosomal protein P0, *Rplp0*,^43^ exactly followed this random distribution model (Fig. 5C). Additionally, as an index of the long right tail of the gene expression distribution, we calculated the skewness of each gene by using the expression levels, which were normalized within the JG cell fraction (Supplementary Fig. S8D). Despite the lower skewness of the *Rplp0* gene (0.6), or that of other housekeeping genes, the skewness of the *Ren* gene was far higher at 6.1, which was one of the largest scores in the top 5% among all of the genes whose average JG-normalized expression was higher than 0.01 (Supplementary Table S9).

Furthermore, we selected the top 25% of all JG cells/spots with the highest normalized *Ren* expression levels from each sample as the ‘high-responding’ spots (Fig. 5D and Supplementary Fig. S8E). For these cells/spots, we calculated the differentially expressed genes (Supplementary Table S10). In addition to the *Ren* gene itself, several other genes appeared to be characteristic of these cells/spots. These genes are associated with those involved in vascular endothelial functions, such as *Tms4sf*^44^ or Emcn.^45^ Consistently, GO analysis showed that GO terms such as ‘circulatory system process’ or ‘blood vessel development’, were associated with the ‘high-responding’ JG cells/spots (Fig. 5E), and the expression of genes involved in the GO term ‘circulatory system process’ indeed increased in the ‘high-responding’ JG cells/spots^46–50^ (Supplementary Fig. S8F). These results collectively suggest that endothelial activation or improved circulation in the high-responding glomeruli may be associated with the observed hyper-responsiveness of these cells. In the high-responding glomeruli, a stronger induction of blood pressure may occur as an effect of the losartan treatment and may further generate feedback for hypotensive responsiveness.

## 4. Discussion

In this study, we constructed a comprehensive catalogue for rat renal cells with spatial information by integrating the scRNA-seq and ST-seq datasets. As comprehensive information regarding the rat kidney has been limited, data from scRNA-seq alone, and losartan, which was administered in this study, is important for both basic and clinical research on the kidney, the datasets we acquired here is worthwhile as a reference dataset for further research on the kidney. In this study, we also performed a cross-species comparative analysis with rat. In particular, another model animal, mouse, showed a high correlation with rat in most of the cell types and histological regions (Supplementary Fig. S1F and S3A). Regarding the podocytes which are specifically located in the glomeruli, the correlation of its expression profile between rat and mouse was relatively low, and the cortical region also showed a relatively low correlation compared with other regions, thus the species difference possibly exists in the cortical region, particularly glomeruli. Consistently, it was previously reported that the glomerular size relative to total kidney weight of the mouse is smaller than that of other animals including that of the rat.^51^ However, when we compared the datasets between rats and humans,^32^ we found the correlation was low (*r* < 0.1; Supplementary Fig. S3B). We consider that this low correlation between rat and human may partially have aroused from the fact that the human snRNA-seq dataset was originally acquired from a different platform rather than the species difference. In this platform, single nuclei were isolated, and library was constructed based on the 5′-capturing method. In addition, the human kidney samples are generally obtained during nephrectomy, thus the background of patients or the sampling region is uneven.

Integration of scRNA-seq with ST-seq revealed differences in the transcriptome depending on the positioning of the cells. Ransick *et al*.,^16^ have attempted the spatial characterization of the nephrotic epithelial cells by scRNA-seq with a specific sampling approach using mice. In that report, they identified the transcriptional differences in DTL sub-populations depending on whether they are a part of cortical nephron which is derived from a cortical glomerulus or the other juxtamedullary nephron as well as their spatial distribution. In this study, we observed that there are transcriptomic differences among DTL sub-populations depending on their spatial distribution estimated by *in silico* integration approach (Fig. 3C) which were consistent with the previous knowledge from mouse kidney with experimental zonation. These results strongly support that the present approach to complement the limited resolution of ST-seq by integration with the corresponding scRNA-seq dataset works well. Additionally, we found that CDPC and CDICa were divided into two sub-populations in the scRNA-seq dataset, and they were also characterized by the spatial positioning in the cortical or medullary region (Fig. 3D and E). Subsequently, we observed the different characteristics of CDICa sub-populations between cortical and medullary regions via GO analysis (Fig. 3F). Although Ransick *et al*.,^16^ also described the spatial diversity of collecting duct cells reflecting their development process, they have not discussed the functional differences involved in the ischaemic state or ammonium transport observed here. However, these spatially distinct characterizations are reasonable by considering the renal anatomy^37^ and consistent with the previous knowledge,^38^ thus the differentially expressed genes identified here aided the characterization of spatial positioning and may still harbour additional information. In comparison with humans, Lake *et al*.,^39^ have also described the spatially distinct profiles of collecting duct by snRNA-seq with experimental zonation. In that study, they characterized the CDICa sub-populations with different spatial distribution by GO analysis like this study, but their results did not include the GO terms enriched in our result. However, they reported the marker genes for cortical or medullary CDPC sub-populations and these marker genes showed expression patterns corresponding with rats (Supplementary Fig. S5C). Furthermore, in another study, the human snRNA-seq dataset was also utilized as a reference for the integration analysis with ST-seq dataset from humans.^23^ Despite the limited collection of medullary compartments in human sample for ST-seq, the imputed distribution for the cortical or medullary CDPC was consistent with the original snRNA-seq zonation. Regarding CDICb which was identified as a single cluster even after unsupervised clustering, they were exclusively enriched in the cortical region, consistent with the previous reports with mice as well as rats.^16^^,^^52–54^ Of note, since the spatial differences were observed even in the results from unsupervised clustering of the scRNA-seq dataset, we may find similar spatial information in the archived scRNA-seq dataset of the kidney.

We further characterized two minor cell populations, such as macula densa and JG cells. Although in a previous study using murine kidney, these minor cell types, in particular macula densa, have been represented in scRNA-seq with a specific FACS-based targeted approach,^40^ our present approach of integrating the ST-seq and scRNA-seq datasets also enabled the characterization of these minor cell populations in conjunction with the spatial orchestration. Furthermore, we also identified the transcriptomic responses to losartan treatment in these minor populations in accordance with the known effect of losartan.^55^ Although the mechanism of action of losartan itself was biologically well known (Fig. 4H), the fact that we observed similar results in our dataset supports the validity of our approach. Thus, we proposed a conceptual scheme to evaluate the drug responses in a spatially resolved manner even in minority populations.

Next, when we considered the Ren expression as an index of the response to losartan, we found spatial heterogeneity in the response among the glomeruli. Indeed, the gene signature specific to the ‘high-responding’ glomeruli was associated with improved circulation or endothelial activation, consistent with the hypertensive action of renin. Afferent arterioles entering every glomerulus are branched from thicker arteries, such as the arcuate artery. The locations of the glomeruli in the cortex are different; thus, the distance from the thicker arteries also differs. These differences in the orientation of blood vessels may affect drug delivery via circulation. Several reports have suggested the heterogeneous drug delivery to tumours with a complex microenvironment via various approaches, and that this is mainly caused by the complicated and heterogeneous microvasculature.^56^^,^^57^ Kidneys also form heterogeneous microvasculature; thus, heterogeneous drug delivery similar to that in tumours might be observed even in healthy conditions. Therefore, the evaluation of whether the drug uniformly reaches the target tissue is critical for the development of drugs with more effective action, and this study presents an approach for the evaluation of the spatial heterogeneity in drug delivery.

In this study, as the cryosections were supplied for ST-seq analysis, it was difficult to annotate the detailed histological structure, such as the difference between the proximal and DTs in the cortex. A bidirectional approach for annotation using both histological and transcriptomic features will achieve a more precise atlas. Recently, another ST-seq platform supporting formalin-fixed paraffin-embedded samples has been launched only for human or mice.^58^ This platform may be useful in obtaining histological images more clearly. In addition, we observed the spatial heterogeneity in drug response, although the region to be analysed was limited and two-dimensions; thus, careful consideration is required because a limited number of glomeruli was included, and the vascular pole where the renin was mainly produced by JG cells was unevenly present in a section. These limitations also made it difficult for us to identify the biased spatial distribution of ‘high-responding’ glomeruli. Nonetheless, this study showed a scheme for spatially resolved pharmacological evaluation using scRNA-seq and ST-seq datasets simultaneously, in an integrated manner.

## Supplementary data

Supplementary data are available at *DNARES* online.

## Supplementary Material

dsac007_Supplementary_Data
