# Supplementary material for: Spatial and single-cell transcriptome analysis reveals changes in gene expression in response to drug perturbation in rat kidney
Source: DNA Res. 2022 Mar 23;29(2):dsac007. doi: 10.1093/dnares/dsac007 (PMC9014450; doi:10.1093/dnares/dsac007)
Supplement: dsac007_Supplementary_Data [file dsac007_supplementary_data.zip › SupplementaryInformation.docx]

**Supplementary Fig. 1:**

**(A, B)** Hematoxylin & Eosin staining images **(A)** and spots colored using unsupervised clustering on histological images **(B)** of each rat treated with losartan (Losa1-3) or untreated (Ctrl1-3). Broken lines show the corresponding area of histological annotations, C: cortex, OMOS: outer medulla outer stripe, OMIS: outer medulla inner stripe, and IM: inner medulla. **(C)** Projections of UMAP dimensional reduction, colored by the histological annotations of each rat. **(D)** Proportion of unsupervised clusters in each rat. **(E, F)** Scatter plots for the comparison of gene expressions between rats and humans **(E)** or mice **(F)** in every corresponding histological region. The gene expression from rat are indicated on the x- axis and the gene expression from humans or mice are indicated on y-axis in log10-scale. Pearson’s correlation coefficients were calculated independently in every histological region.

**Supplementary Fig. 2:**

**(A)** Expression of the *C1qc* gene, a marker gene for macrophages, in each immune cell type population. **(B-D)** Projections of UMAP dimensional reduction colored according to the sub-populations (left) and the proportion of the subpopulations (right) present in Epithelial **(b)**, Immune **(C)**, and Stromal **(D)** population in each rat. The spots colored grey in UMAP projections are cells representing contamination from other populations and are not included in the bar graph depicting proportions.

**Supplementary Fig. 3:**

Scatter plots for the comparison of gene expression between rat and mouse **(A)** or human **(B)** in every corresponding cell type. The gene expression from rat are indicated on the x- axis and the gene expression from mouse or human is indicated on the y-axis in log10-scale. Pearson’s correlation coefficients were calculated independently in every cell type, podocyte (Podo), proximal tubule (PT), loop of Henle (LOH), thick ascending limb (TAL), distal convoluted tubule (DCT), connecting tubule (CNT), and collecting duct principal cells (CD-PC or CDPC) or intercalated cells (CD-IC, CDIC-A; type A, and CDIC-B; type B for human).

**Supplementary Fig. 4:**

**(A)** Predicted scores of each cell type in the Epithelial population from the scRNA-seq data (x-axis) for each unsupervised cluster. The ST-seq data (y-axis) are shown, separated by histological annotation. The color of the dots represents the scaled predicted scores, and the size of the dots represents the percentage of the spots with a predicted score which is larger than 0 in each histological annotation. **(B)** The distribution of the predicted scores for PT and the four subpopulations of DTL in each histological annotation. **(C)** The distribution of the predicted scores for the four subpopulations of DTL (left), and expression of representative genes among the significantly expressed genes for each subpopulation (right) on a histological image from a control rat. Dashed white lines show the corresponding histological annotations. **(D, E)** Predicted scores of each cell type among the Immune **(D)** and Stromal **(E)** populations from the scRNA-seq data (x-axis) for each unsupervised cluster using ST-seq data (y-axis).

**Supplementary Fig. 5:**

**(A, B)** The distribution of the predicted scores for the two subpopulations of CDPC **(A)** and CDICa **(B)** on a histological image from a control rat (top). Dashed white lines show the corresponding histological annotations. Expression of representative genes among the differentially expressed genes for each subpopulation of CDPC **(A)** and CDICa **(B)** in each histological annotation (bottom). **(C)** Expression of the representative marker genes for CDICb on a histological image from a control rat (left) and in each histological annotation (right). Dashed white lines show the cortical area in which the CDICb predicted score was highly enriched.

**Supplementary Fig. 6:**

**(A)** Histological image showing the area near a glomerulus in each rat. Dashed white circles show the spots where predicted scores for TAL-2 are higher than 0.8. **(B)** The distribution of the predicted scores for the two subpopulations of TAL in each histological annotation. **(C)** Expression of *Pappa2* in each cell type of the Epithelial population. **(D)** Heatmap showing the expression of the top 10 differentially expressed genes between the rats treated with losartan (Losa) or untreated (Ctrl) in the TAL-2 subpopulation of scRNA-seq. **(E)** The fraction of TAL-2 in each spot imputed by CIBERSORTx deconvolution. Red line indicates the fraction value for 0.02 which was used for subset of macula densa spots. **(F)** The fraction of Epithelial population imputed by CIBERSORTx in 374 macula densa spots. Each column indicates a single spot in ST-seq. **(G)** Expression of the *Slc12a1* gene, a representative gene among the differentially expressed genes between the Losa and Ctrl groups, as calculated using the expression value normalized by TAL-2 fraction. Statistical analysis was performed by Wilcoxon rank sum test, and calculated p-values are shown.

**Supplementary Fig. 7:**

**(A)** Heatmap showing the expression of the top 10 differentially expressed genes between the rats treated with losartan (Losa) or untreated (Ctrl) in the Peri-Ren+ subpopulation of scRNA-seq. **(B)** The fraction of Peri-Ren+ in each spot of cortex, imputed by CBERSORTx deconvolution. Red line indicates the fraction value for 0.02 which was used to subset juxtaglomerular cell spots. **(C)** The fraction of Stromal population imputed by CIBERSORTx in selected juxtaglomerular cell spots. Each column indicates a single spot in ST-seq. **(D)** Expression of the Ren gene in Losa and Ctrl groups in the unsupervised cluster 10 in ST-seq, which is presumed to be the spots overlying the glomeruli. Statistical analysis was performed by Wilcoxon rank sum test, and calculated p-values are shown.

**Supplementary Fig. 8:**

**(A)** The selected spots which overlie glomeruli on the histological images of each rat. **(B)** The proportion of the unsupervised clusters in selected glomeruli spots from each rat. **(C)** The fraction of the Stromal population imputed by CIBERSORTx in further selected glomerular cell spots which contain a certain level of Peri-Ren+ fraction. Each column indicates a single spot in ST-seq. **(D)** The distribution of the skewness of single gene expression level, normalized with the imputed fraction of juxtaglomerular cells. Genes whose average expression is lower than 0.01 were filtered out. The blue and red line indicate a housekeeping gene, *Rplp0*, and *Ren*, respectively. **(E)** The distribution of Ren gene expression levels normalized with the juxtaglomerular cell fraction from all three rats treated with losartan. The red line indicates the third quartile, and the upper 25% of spots were assigned as the high-responding glomeruli spots. **(F)** The expression levels of several genes included in the GO term “circulatory process” (*Gja5*, *Tnnc1*, *Tpm1* or *Cav1*) in selected glomeruli spots, and separated into “high-responding” spots (high) and other (low). Statistical analysis was performed by Wilcoxon rank sum test, and calculated p-values are shown.

**Supplementary Table 1: General statistics of ST-seq data**

| Sample ID | Ctrl1 | Ctrl2 | Ctrl3 | Losa1 | Losa2 | Losa3 |
| --- | --- | --- | --- | --- | --- | --- |
| Number of Spots Under Tissue | 2573 | 2526 | 2782 | 3008 | 3223 | 2316 |
| Median Genes per Spot | 4959 | 5434 | 5476.5 | 4843 | 4918 | 5236.5 |
| Number of Reads | 5.01E+08 | 4.33E+08 | 4.92E+08 | 4.17E+08 | 4.67E+08 | 4.14E+08 |
| Valid Barcodes | 0.965 | 0.964 | 0.964 | 0.964 | 0.963 | 0.963 |
| Valid UMIs | 0.999 | 0.999 | 0.999 | 0.999 | 0.999 | 0.999 |
| Mean Reads per Spot | 194525.5 | 171519.8 | 176915.5 | 138715.3 | 144783.7 | 178554.1 |
| Sequencing Saturation | 0.791 | 0.704 | 0.734 | 0.746 | 0.724 | 0.730 |
| Q30 Bases in Barcode | 0.946 | 0.948 | 0.949 | 0.946 | 0.943 | 0.942 |
| Q30 Bases in RNA Read | 0.925 | 0.925 | 0.925 | 0.923 | 0.916 | 0.919 |
| Q30 Bases in UMI | 0.943 | 0.944 | 0.946 | 0.943 | 0.940 | 0.939 |
| Reads Mapped to Genome | 0.913 | 0.909 | 0.941 | 0.919 | 0.931 | 0.938 |
| Reads Mapped Confidently to Genome | 0.852 | 0.848 | 0.875 | 0.861 | 0.872 | 0.877 |
| Reads Mapped Confidently to Intergenic Regions | 0.114 | 0.115 | 0.113 | 0.118 | 0.112 | 0.110 |
| Reads Mapped Confidently to Intronic Regions | 0.022 | 0.023 | 0.022 | 0.023 | 0.021 | 0.021 |
| Reads Mapped Confidently to Exonic Regions | 0.716 | 0.710 | 0.741 | 0.720 | 0.739 | 0.747 |
| Reads Mapped Confidently to Transcriptome | 0.685 | 0.678 | 0.711 | 0.689 | 0.708 | 0.716 |
| Reads Mapped Antisense to Gene | 0.006 | 0.006 | 0.007 | 0.007 | 0.007 | 0.007 |
| Fraction Reads in Spots Under Tissue | 0.951 | 0.896 | 0.927 | 0.924 | 0.948 | 0.921 |
| Total Genes Detected | 16699 | 16985 | 16924 | 16668 | 16864 | 16685 |
| Median UMI Counts per Spot | 24246 | 28131 | 29344.5 | 21457 | 24363 | 29595.5 |
| Fraction of Spots Under Tissue | 0.515 | 0.506 | 0.557 | 0.603 | 0.646 | 0.464 |

**Supplementary Table 2: General statistics of scRNA-seq data**

| Sample ID | Ctrl | Losa |
| --- | --- | --- |
| Estimated Number of Cells | 7,284 | 7,546 |
| Mean Reads per Cell | 46,610 | 44,341 |
| Median Genes per Cell | 1,137 | 1,141 |
| Number of Reads | 339,507,703 | 334,603,296 |
| Valid Barcodes | 98.50% | 98.40% |
| Sequencing Saturation | 78.50% | 79.60% |
| Q30 Bases in Barcode | 97.60% | 97.60% |
| Q30 Bases in RNA Read | 65.80% | 74.40% |
| Q30 Bases in UMI | 97.80% | 97.80% |
| Reads Mapped to Genome | 89.60% | 93.80% |
| Reads Mapped Confidently to Genome | 83.30% | 87.70% |
| Reads Mapped Confidently to Intergenic Regions | 11.60% | 14.10% |
| Reads Mapped Confidently to Intronic Regions | 8.50% | 9.70% |
| Reads Mapped Confidently to Exonic Regions | 63.30% | 64.00% |
| Reads Mapped Confidently to Transcriptome | 59.90% | 61.30% |
| Reads Mapped Antisense to Gene | 1.30% | 0.50% |
| Fraction Reads in Cells | 86.80% | 87.60% |
| Total Genes Detected | 17,369 | 16,791 |
| Median UMI Counts per Cell | 3,607 | 3,470 |

**Supplementary Table 6: The number of TAL spots adjacent to the glomeruli**

|  | TAL-1 | TAL-2 |
| --- | --- | --- |
| False | 1939 | 1303 |
| True | 707 | 1212 |

*Fisher’s exact test for count data; p-value < 2.2e-16, odds ratio = 3.01531

**Supplementary Table 8: The number of responding spots histologically overlapped with glomeruli**

|  | Control | Losartan |
| --- | --- | --- |
| Non-responding | 137 | 54 |
| Responding | 386 | 512 |

*Fisher’s exact test for count data; p-value = 3.447e-13, odds ratio = 3.361455

The supplemental tables below are supplied by another file in Excel format.

**Supplementary Table 3: The specific genes calculated for each cell type**

**Supplementary Table 4: The specific genes calculated among DTL subpopulations**

**Supplementary Table 5: Differentially expressed genes calculated between CDPC and CDICa subpopulations**

**Supplementary Table 7: The specific genes calculated for the TAL-2 subpopulation**

**Supplementary Table 9: The degree of skewness for gene expressions normalized using the JG fraction**

**Supplementary Table 10: Differentially expressed genes calculated between high-responding and other glomeruli spots**
