# Supplementary figures and images for: Spatial and single-cell transcriptome analysis reveals changes in gene expression in response to drug perturbation in rat kidney
Source: DNA Res. 2022 Mar 23;29(2):dsac007. doi: 10.1093/dnares/dsac007 (PMC9014450; doi:10.1093/dnares/dsac007)

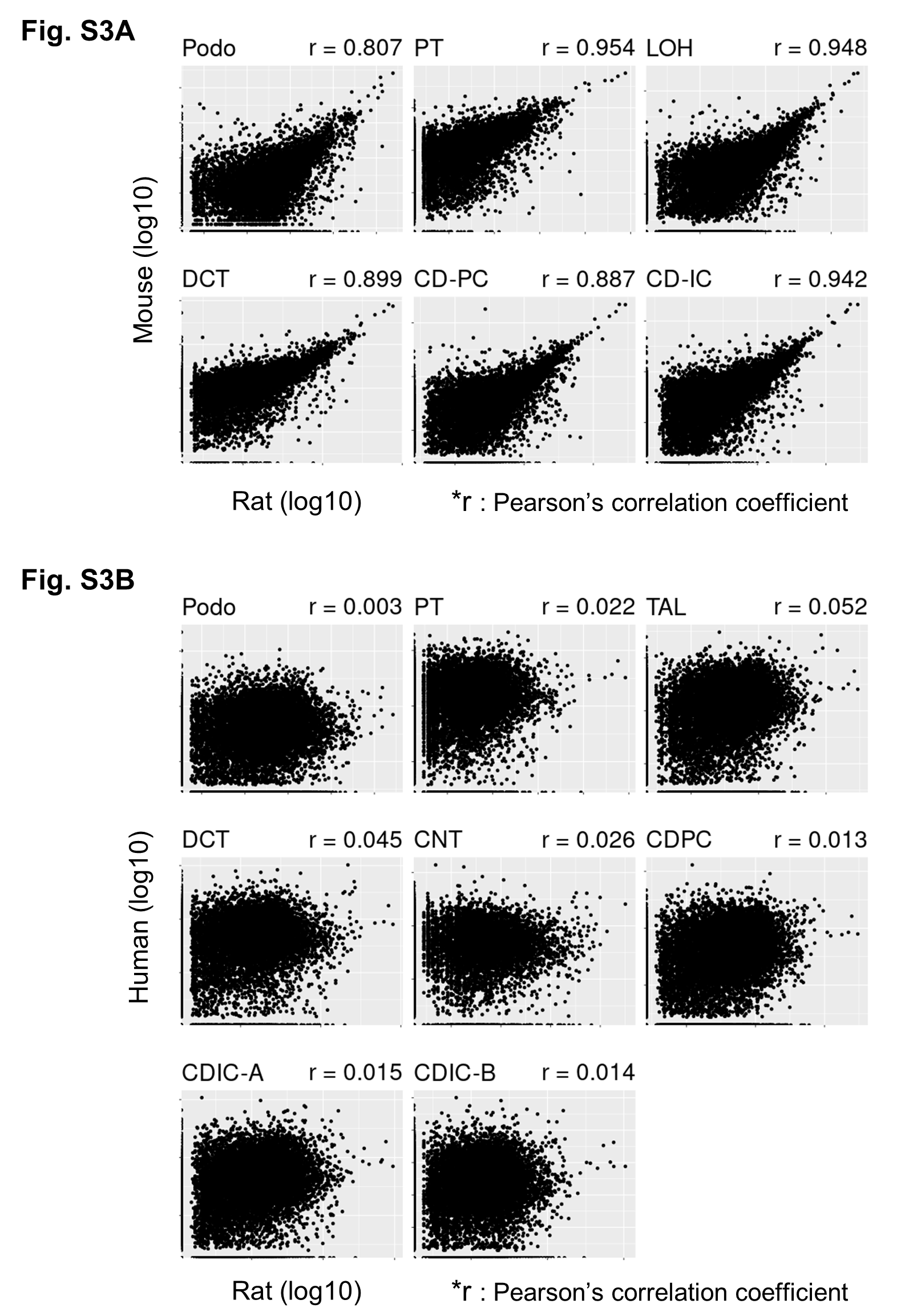

Supplement: dsac007_Supplementary_Data [file dsac007_supplementary_data.zip › FigS3.TIF]

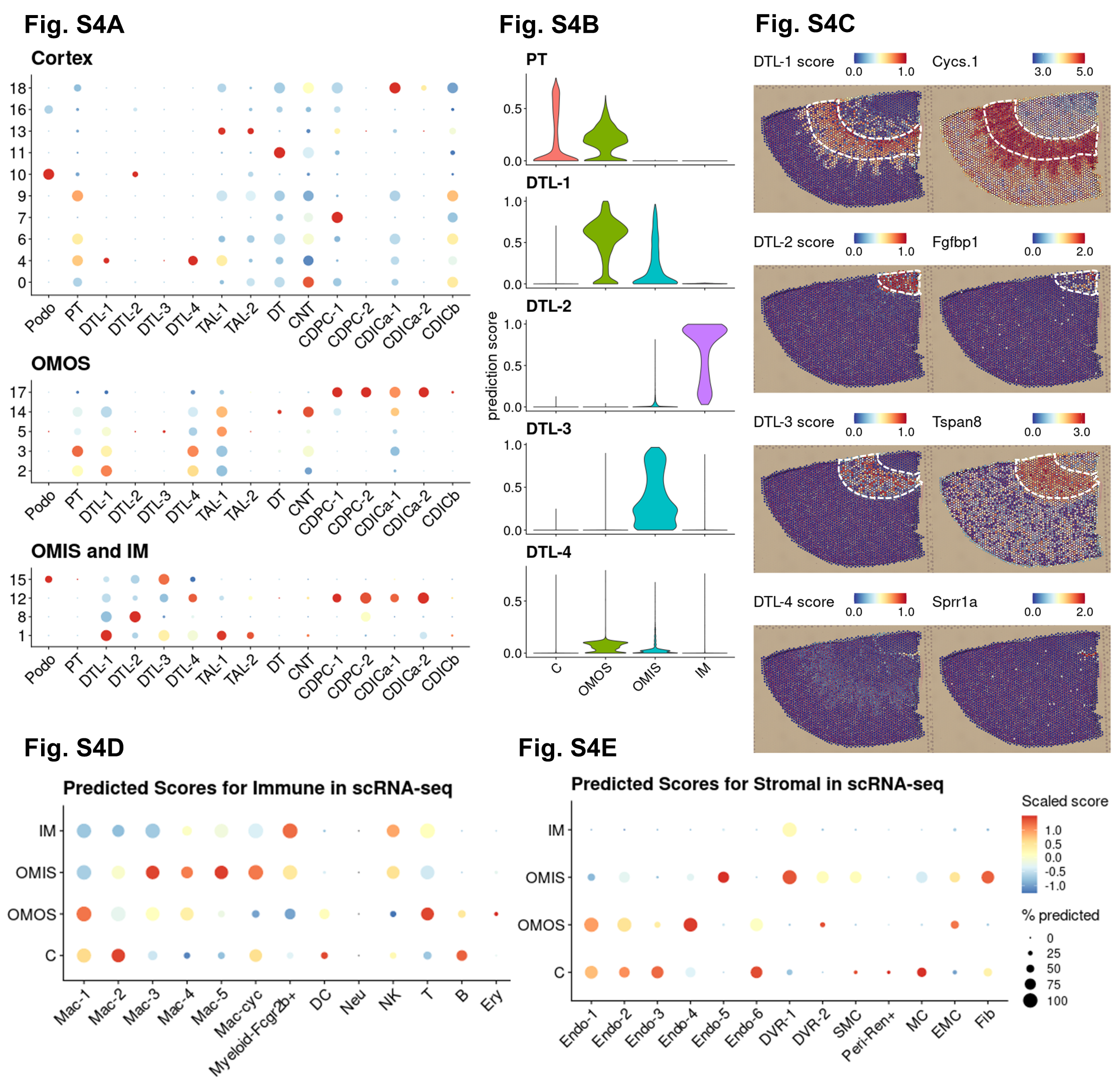

Supplement: dsac007_Supplementary_Data [file dsac007_supplementary_data.zip › FigS4.TIF]

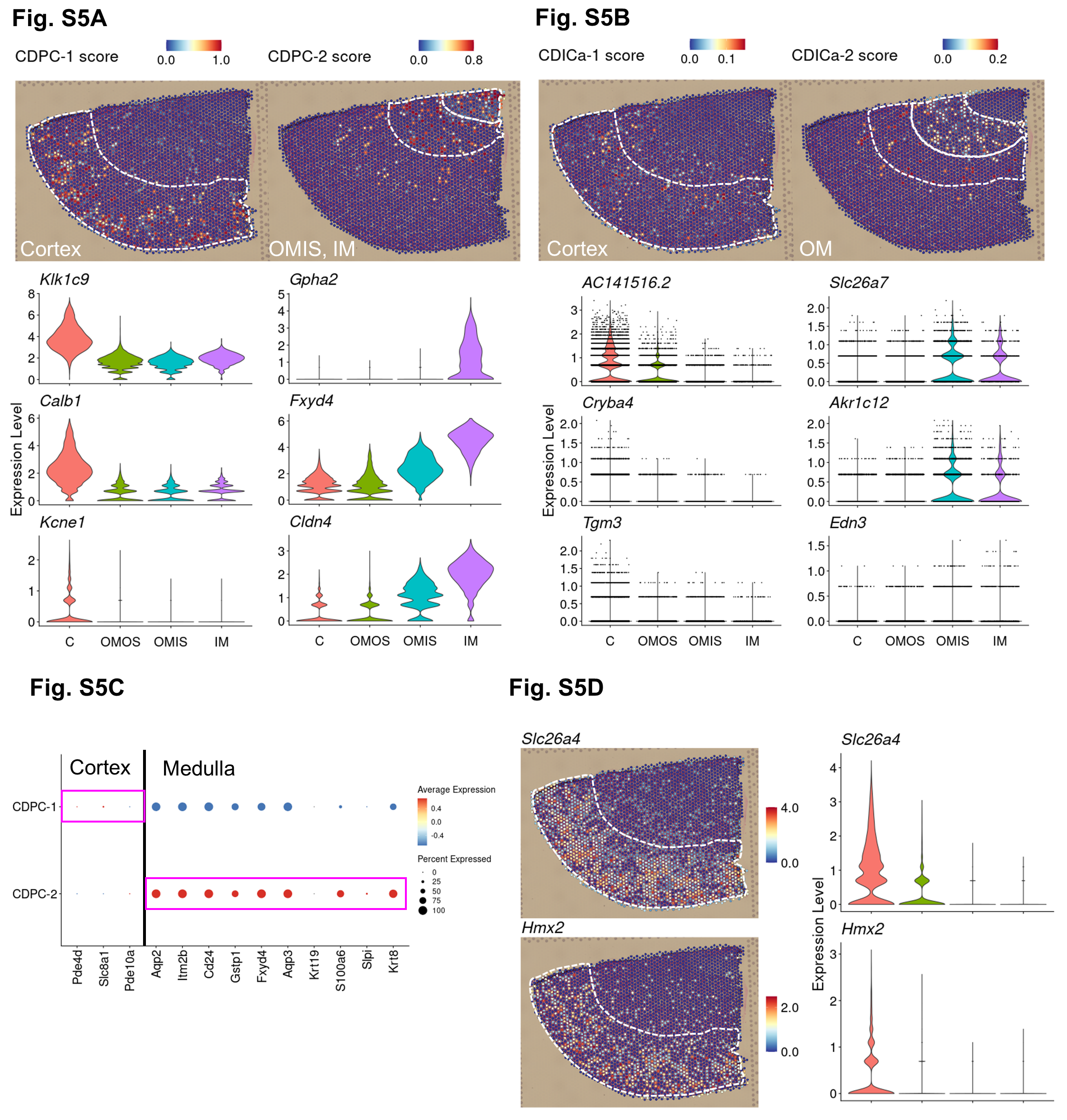

Supplement: dsac007_Supplementary_Data [file dsac007_supplementary_data.zip › FigS5.TIF]

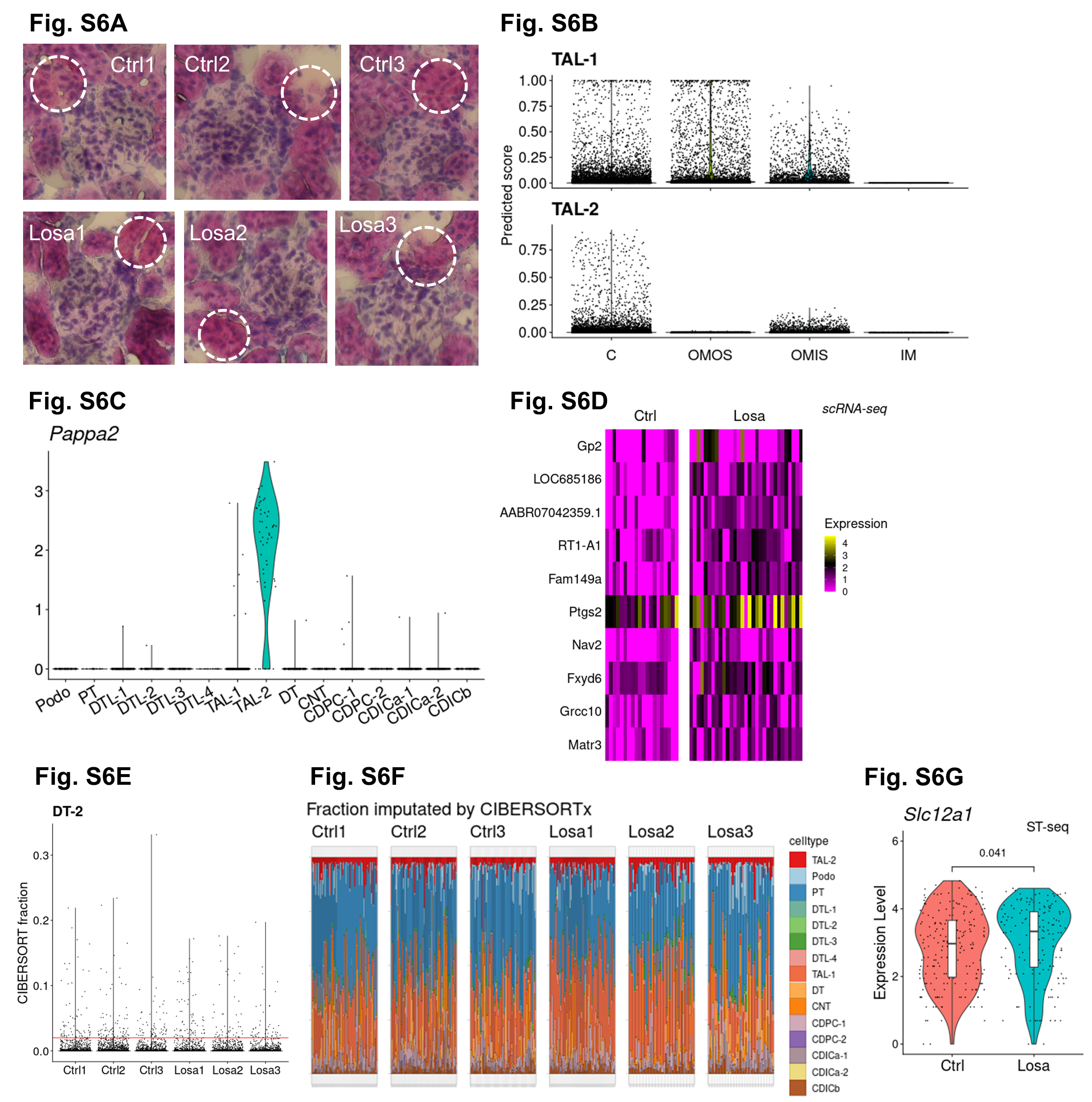

Supplement: dsac007_Supplementary_Data [file dsac007_supplementary_data.zip › FigS6.TIF]

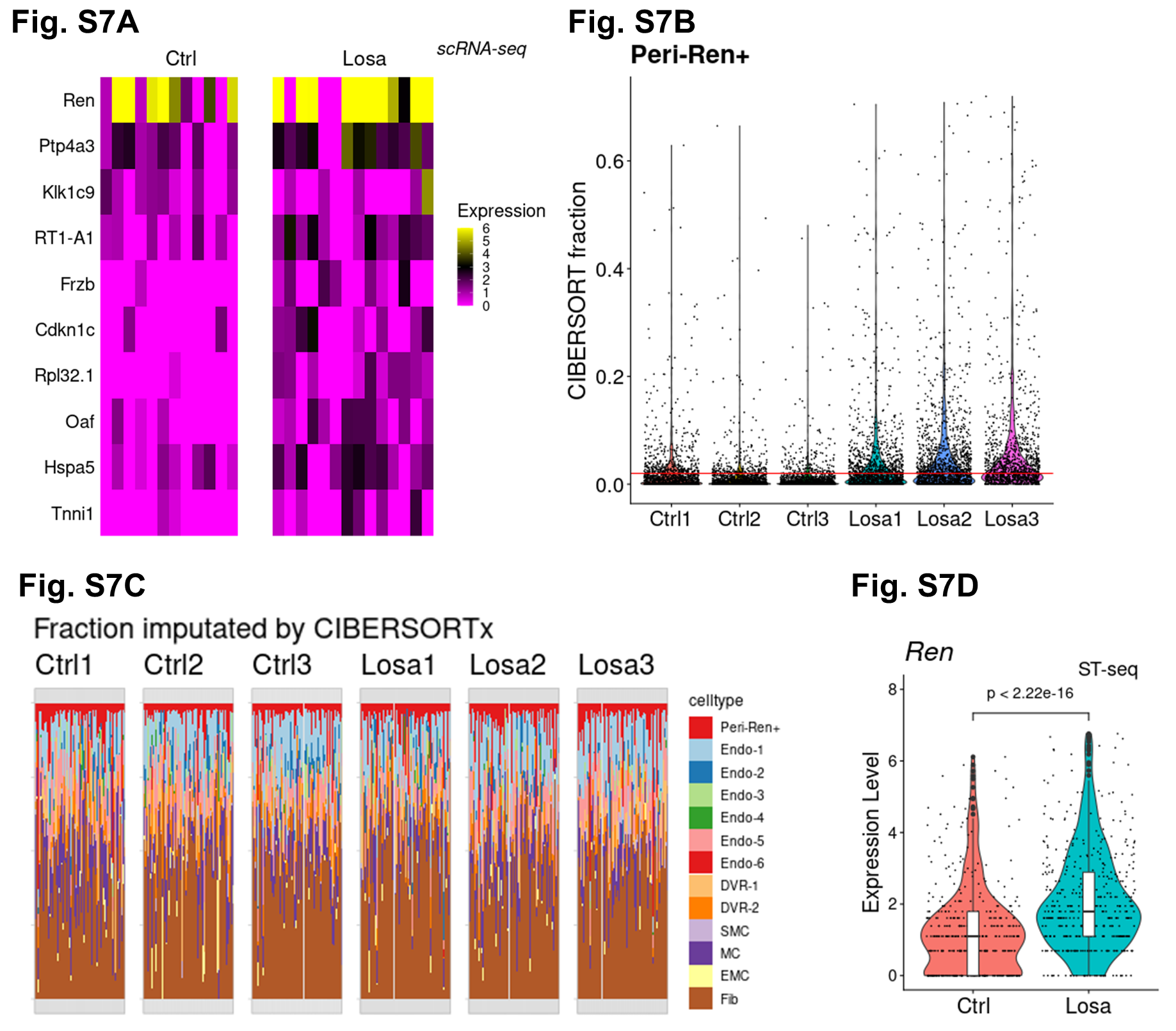

Supplement: dsac007_Supplementary_Data [file dsac007_supplementary_data.zip › FigS7.TIF]

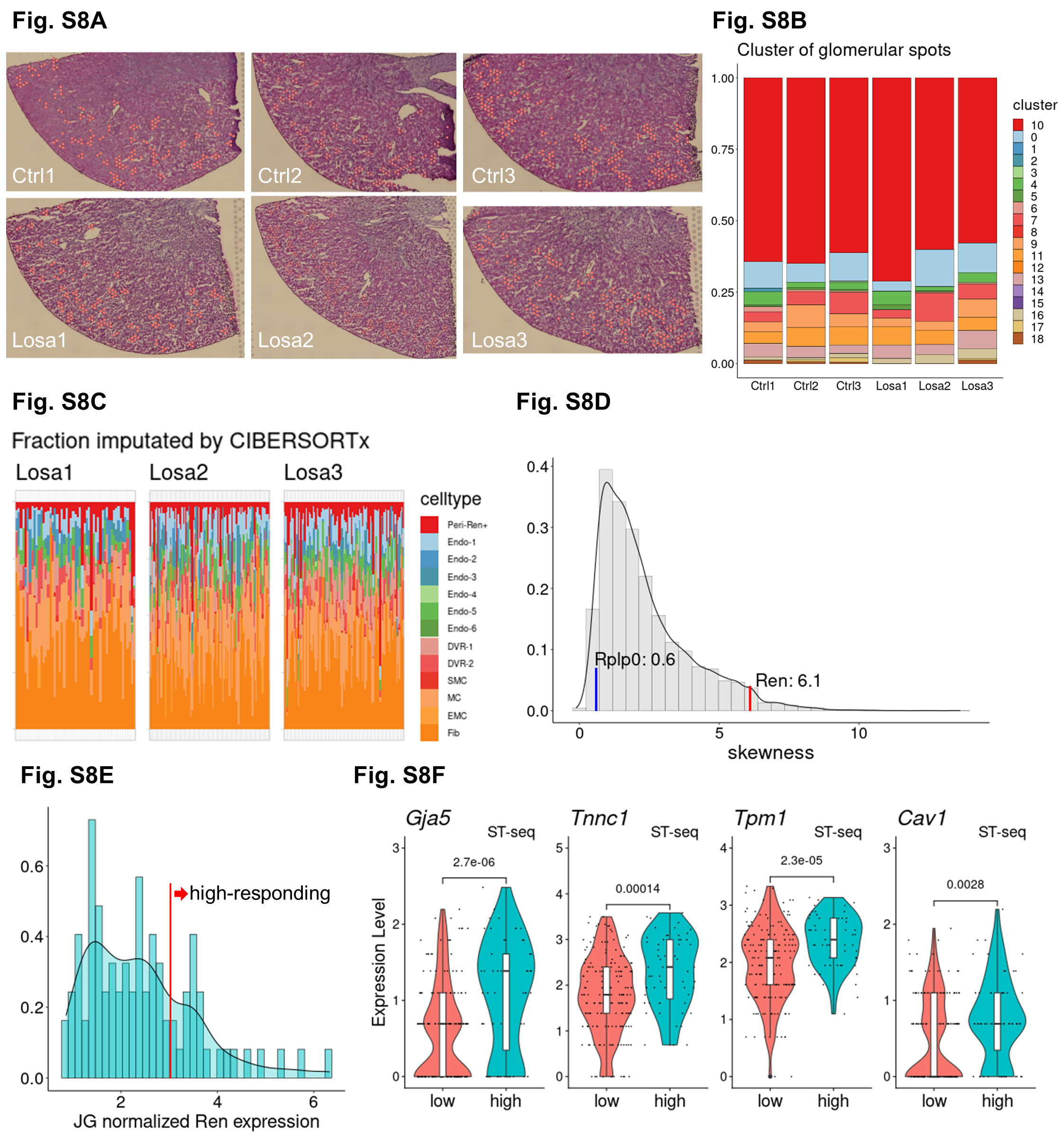

Supplement: dsac007_Supplementary_Data [file dsac007_supplementary_data.zip › FigS8.TIF]

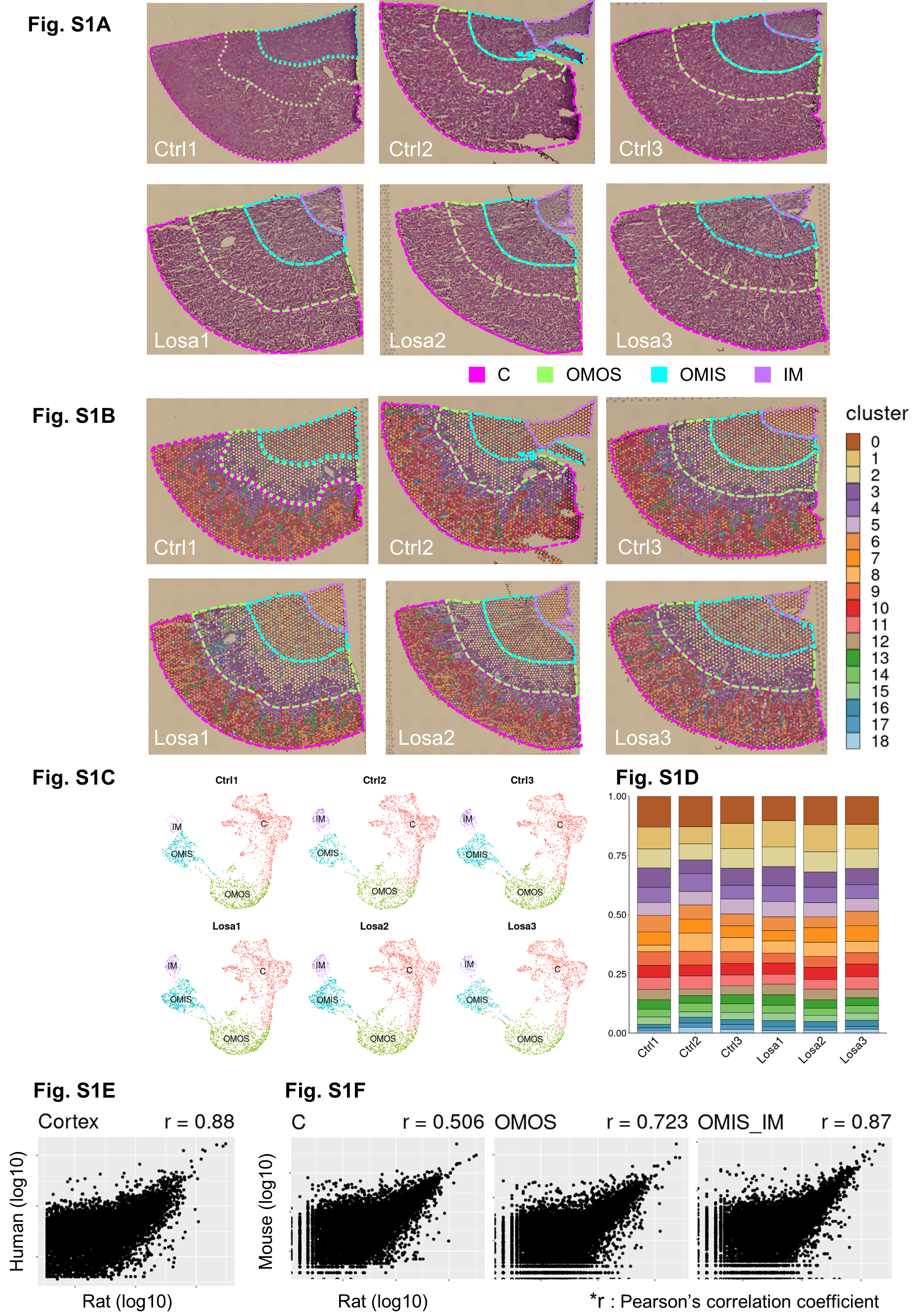

Supplement: dsac007_Supplementary_Data [file dsac007_supplementary_data.zip › FigS1.TIF]

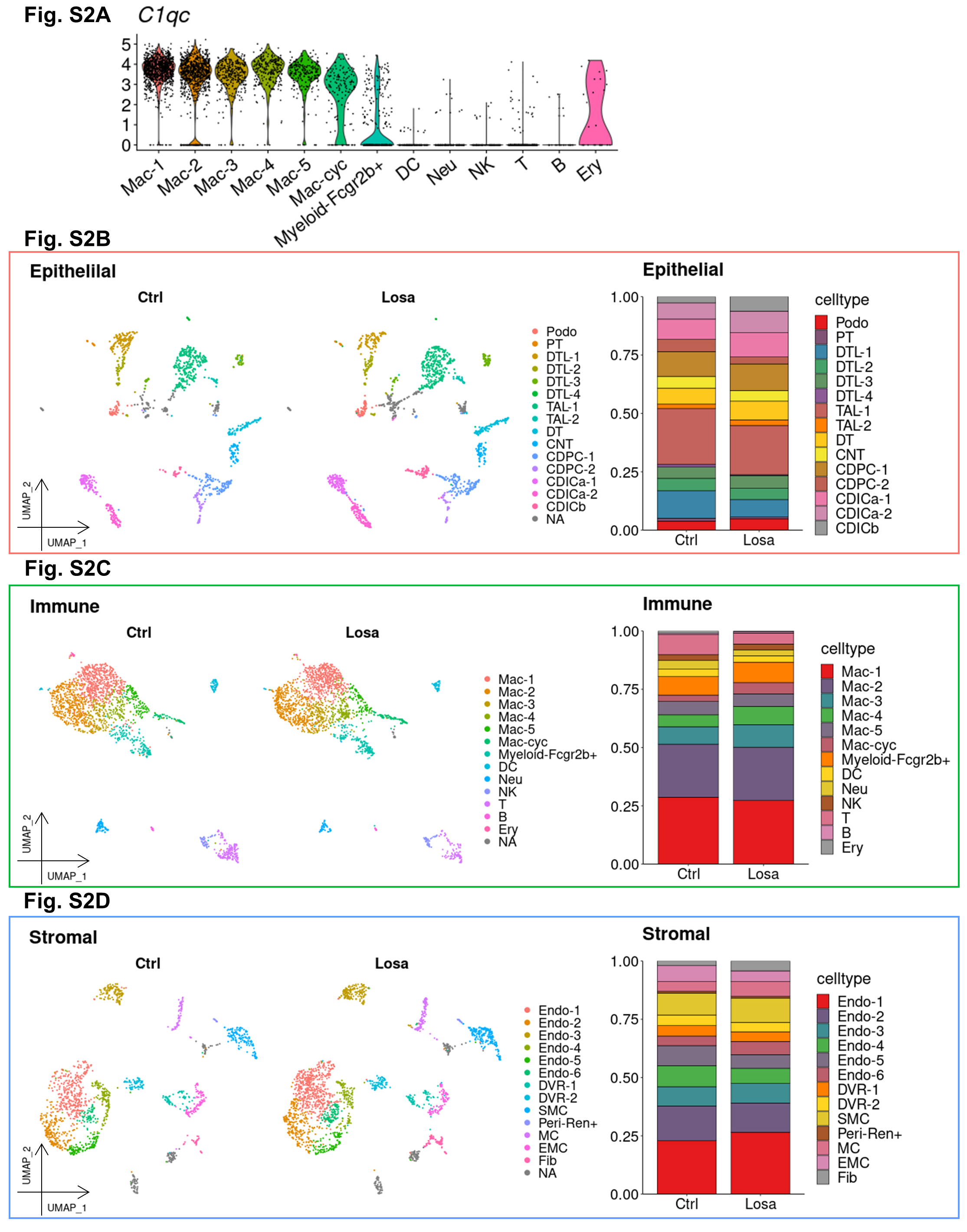

Supplement: dsac007_Supplementary_Data [file dsac007_supplementary_data.zip › FigS2.TIF]
